# Self-Collection for Cervical Cancer Screening in a Safety-Net Setting: The PRESTIS Randomized Clinical Trial

**DOI:** 10.1001/jamainternmed.2025.2971

**Published:** 2025-06-06

**Authors:** Jane R. Montealegre, Susan G. Hilsenbeck, Shaun Bulsara, Susan L. Parker, Trisha L. Amboree, Matthew L. Anderson, Maria Daheri, Maria L. Jibaja-Weiss, Kathleen M. Schmeler, Ashish A. Deshmukh, Elizabeth Y. Chiao, Michael E. Scheurer

**Affiliations:** 1 Department of Behavioral Science, The University of Texas MD Anderson Cancer Center, Houston; 2 Department of Medicine, Baylor College of Medicine, Houston, Texas; 3 Dan L Duncan Comprehensive Cancer Center, Baylor College of Medicine, Houston, Texas; 4 Department of Public Health Sciences, College of Medicine, Medical University of South Carolina, Charleston; 5 Cancer Prevention & Control Program, Hollings Cancer Center, Medical University of South Carolina, Charleston; 6 Department of Obstetrics and Gynecology, Morsani College of Medicine, University of South Florida, Tampa; 7 Harris Health System, Houston, Texas; 8 School of Health Professions, Baylor College of Medicine, Houston, Texas; 9 Department of Gynecologic Oncology, The University of Texas MD Anderson Cancer Center, Houston; 10 Department of Epidemiology, The University of Texas MD Anderson Cancer Center, Houston; 11 Center for Epidemiology and Population Health, Department of Pediatrics, Baylor College of Medicine, Houston, Texas

## Abstract

**Importance:**

There are limited data on the effectiveness of mailed self-collection to increase cervical cancer screening (CCS) participation in underresourced health care settings.

**Objective:**

To compare the effectiveness of mailed self-collection kits, with and without patient navigation, to telephone reminders to increase CCS in a safety-net health system.

**Design, Setting, and Participants:**

This pragmatic, parallel, single-blinded, randomized clinical trial within a publicly funded safety-net health system in Houston, Texas, compared (1) telephone reminder (TR) for clinic-based screening, (2) TR with mailed self-collection (SC), and (3) TR with mailed SC and patient navigation among a random sample of CCS-eligible patients not up to date with CCS, including those with no CCS on record. The trial was conducted from February 20, 2020, to August 31, 2023.

**Interventions:**

All groups received a TR by a patient navigator to attend clinic-based CCS. In the SC and SC with patient navigation groups, participants were additionally mailed a self-collection kit to their home as an alternative to clinic-based CCS. In the SC with patient navigation group, the mailed kit was followed by a patient navigation telephone call.

**Main Outcomes and Measures:**

CCS participation was defined as attendance for clinic-based screening or return of a mailed self-collection kit within 6 months of randomization and determined through electronic health record review.

**Results:**

Of the 2474 participants in the intent-to-screen analyses (median [IQR] age, 49 [39-57] years), 2325 (94.0%) were from racial or ethnic minoritized populations (1655 [66.9%] identifying as Hispanic or Latino, 82 [3.3%] as non-Hispanic Asian, 535 [21.6%] as non-Hispanic Black or African American, and 53 [2.1%] as other or unknown race, including American Indian or Alaska Native and Native Hawaiian or Other Pacific Islander), and 1388 (56.1%) were covered by the county’s publicly funded financial assistance program. At 6 months, 144 of 828 participants (17.4%) in the TR group, 340 of 828 (41.1%) in the SC group, and 381 of 818 (46.6%) in the SC with patient navigation group had participated in CCS. Compared to TR, relative participation was 2.36 (95% CI, 1.99-2.80) times higher for SC and 2.68 (95% CI, 2.27-3.16) times higher for SC with patient navigation; screening difference was 23.7% (95% CI, 19.4%-27.9%) for SC and 29.2% (95% CI, 24.9%-33.5%) for SC with patient navigation.

**Conclusions and Relevance:**

In this randomized clinical trial in a safety-net health system, SC was effective for increasing CCS participation among underscreened patients; there were modest additional gains from SC with patient navigation. The large increase in CCS participation using SC compared to TR suggest that SC should be considered in safety-net settings with suboptimal CCS coverage.

**Trial Registration:**

ClinicalTrials.gov Identifier: NCT03898167

## Key Points

**Question:**

How do mailed self-collection kits, with and without patient navigation, compare to standard telephone reminders to increase cervical cancer screening (CCS) in a safety-net setting?

**Findings:**

In this randomized clinical trial of 2474 individuals overdue for CCS, participants randomized to telephone reminder with mailed self-collection had 41.1% participation compared to those with a telephone reminder alone (17.4%). Participation was modestly increased among those randomized to telephone reminder, mailed self-collection, and patient navigation (46.6%).

**Meaning:**

Mailed self-collection was effective for increasing CCS in a safety-net health setting; additional modest increases were attained by pairing self-collection and patient navigation.

## Teaser

This randomized clinical trial compares the effectiveness of mailed self-collection kits, with and without patient navigation, to telephone reminders to increase cervical cancer screening in a safety-net health system.

## Introduction

Cervical cancer can be eliminated as a public health problem with high population-level coverage of vaccination against human papillomavirus (HPV), timely screening and early detection, and treatment of precancerous lesions.^1^ Estimated projections show that national-level elimination is possible in the US in the next 1 to 2 decades if cervical cancer screening (CCS) coverage were scaled up to 90%.^2^ However, up-to-date CCS participation has declined in recent years^3^ (75.2% in 2021^4^) to below the Healthy People 2030 target of 79.2%.^5^ Furthermore, CCS rates are notably lower among underserved populations, including women and persons with a cervix who are uninsured or publicly insured, live in rural areas, and are from racial or ethnic and sexual or gender minoritized populations.^6^ This suboptimal screening coverage will likely prolong the timeline to national-level elimination^2^ and delay elimination in specific underserved populations by several decades.^7^

Self-collection of vaginal samples for primary HPV testing decreases barriers associated with clinic-based screening involving a pelvic examination (ie, cytology testing and cytology-HPV cotesting^8^) and improves population-level CCS coverage among unscreened and underscreened individuals.^9,10^ Self- and clinician-collected samples have similar accuracy for detection of cervical precancer when tested using polymerase chain reaction assays,^10,11^ and acceptability of self-collection is high.^12^ In May 2024, the US Food and Drug Administration (FDA) approved self-collection for use in health care settings^13^; home-based self-collection is anticipated to receive FDA approval in the near future.^14^ Home-based self-collection using mailed kits is an option in several regional and national screening programs,^15,16^ with demonstrated effectiveness for increasing CCS.^9,10^ However, most pragmatic trials have been conducted in the context of organized CCS programs, predominantly in countries with universal health care coverage.^17,18,19,20,21,22,23,24,25,26,27,28,29,30,31,32,33^ Published pragmatic trials in the US include 2 among insured patients in an integrated managed health system^34,35^ and 1 across multiple health systems in the underserved region of Appalachia.^36^ As self-collection is introduced in the US, data are critically needed to inform implementation in diverse health care settings, particularly in safety-net settings that provide preventive care to the underserved populations who shoulder a disproportionate burden of disease.^37^

Safety-net health systems (ie, those offering access to health care regardless of patients’ ability to pay^38^) are critical settings for the implementation of self-collection in the US.^39^ Safety-net settings provide care to a large proportion of people living in poverty and other underserved populations, including individuals from minoritized racial or ethnic groups.^40^ Cervical cancer incidence is more than 1.5 times higher in individuals from minoritized racial or ethnic groups compared to non-Hispanic White individuals^41^ and 1.5 times higher in those with low vs high income.^42^ Due to limited resources, safety-net settings often have limited ability and capacity to provide CCS services to their patients, resulting in low CCS coverage.^43^ Self-collection addresses many barriers that arise from the need for an in-clinic pelvic examination.^44^ Nonetheless, patients in safety-net settings often face additional barriers (eg, language differences, low health literacy, and distrust in the health system)^45,46^ that may hinder the effectiveness of mailed self-collection. Patient navigation, a patient-centered intervention to promote access to care,^47^ can be used to address these barriers^48^ and may thus enable participation in self-collection.^36^ The primary objective of the Prospective Evaluation of Self-Testing to Increase Screening (PRESTIS) trial is to evaluate the effectiveness of mailed self-collection to increase screening participation among never- and underscreened patients in a safety-net setting and identify whether self-collection with patient navigation further increases CCS participation.

## Methods

### Trial Design

Underscreened patients in a safety-net health system were randomized to 1 of 3 groups: (1) telephone reminder (TR) by a patient navigator for clinic-based screening; (2) TR with mailed self-collection (SC); and (3) TR, mailed SC, and patient navigation. The trial protocol has been previously published^39^ and is also available in Supplement 1.

Trial enrollment began on February 20, 2020, then paused on March 27, 2020, due to clinic closures and restrictions on human participant research following the declaration of the COVID-19 pandemic. Trial enrollment resumed August 3, 2020, and continued through August 31, 2023.

This study was approved by the institutional review board of Baylor College of Medicine and administratively approved by Harris Health. A waiver of informed consent was granted to identify potentially eligible patients through a query of electronic health records (EHRs). We followed the Consolidated Standards of Reporting Trials (CONSORT) reporting guidelines.

### Study Setting

PRESTIS was embedded in Harris Health, the primary safety-net health care system for Harris County, Texas, and third largest in the US. Standard-of-care CCS involves cytology testing every 3 years for women and persons with a cervix aged 21 to 29 years and cytology-HPV cotesting every 5 years for those aged 30 to 65 years. Opportunistic strategies to promote CCS include EHR flagging of patients due or past due for CCS and patient education. In 2020, CCS coverage in the health system was 68.2%. Pragmatic Explanatory Continuum Indicator Summary 2 criteria were followed to integrate pragmatic elements in the trial design.^39^

### Participants

Women and persons with a cervix aged 30 to 65 years were eligible if they (1) had no history of hysterectomy or cervical cancer, (2) had 2 or more ambulatory care visits in the past 5 years, (3) had no cytology in the past 3.5 years or no HPV test in the past 5.5 years, and (4) were currently enrolled in a health care coverage or financial assistance plan accepted by the health system (ie, Medicaid/Medicare, county publicly funded financial assistance program, state grants, private insurance). Exclusion criteria were (1) no telephone contact information in the EHR, (2) named primary care clinician outside of the health system (which usually indicates referral for specialty care), or (3) documented history of cervical dysplasia in the past 3.5 years. Collected participant sociodemographic and health care characteristics included age, race and ethnicity (Hispanic or Latino, non-Hispanic Asian, non-Hispanic Black or African American, non-Hispanic White, and non-Hispanic other or unknown [including non-Hispanic American Indian or Alaska Native and Native Hawaiian or Other Pacific Islander, which were grouped together owing to small sample sizes]), primary language spoken, tobacco use, health care coverage, and time since last screening.

### Randomization and Masking

Individuals meeting eligibility criteria were contacted by telephone by a patient navigator to deliver TR. After TR, individuals were assessed for additional exclusion criteria (ie, inability to communicate in English or Spanish, self-reported current pregnancy). Eligible individuals were randomized using a permuted-block randomization sequence in REDCap electronic data capture tools (Vanderbilt University) hosted at Baylor College of Medicine.^49,50^ Participants were enrolled under a waiver of consent to reduce participation bias and blinded to study group assignment. Patient navigators delivering the interventions were not blinded to study group assignment; blinded study staff assessed outcomes.

### Interventions

TR involved (1) informing the individual of their need for CCS, (2) CCS education, and (3) invitation to attend clinic-based screening. After the standardized TR script, participants randomized to SC or SC with patient navigation were informed that, as an alternative to clinic-based screening, they would be sent a self-collection kit. They were asked to verify and/or update their mailing address, which they could decline per health system regulations.

Self-collection kits were mailed to participants’ homes within 1 week of TR. Kits included an Aptima Multitest Swab and Specimen Transport Medium (Hologic), an invitation letter on behalf of health system leadership, an illustrated instructional brochure (in English or Spanish based on language of the TR), a research information sheet providing trial information, and a prepaid, return-addressed padded envelope. Participants in the SC with patient navigation group who did not return a kit within 3 weeks of the TR encounter received a second patient navigation telephone call to provide additional instruction and encouragement.

Returned kits were tested in the health system’s Clinical Laboratory Improvement Amendments–certified laboratory using the Aptima HPV test (Hologic) and reflex tested for HPV-16 and HPV-18/45. Results were reported as high-risk HPV negative; high-risk HPV positive and HPV-16, HPV-18, or HPV-45 negative; high-risk HPV positive and HPV-16, HPV-18, or HPV-45 positive; or inadequate due to unsatisfactory sample. Participants were notified of their results by telephone by a patient navigator. Participants with a high-risk HPV positive test were referred for clinical follow-up according to the American Society for Colposcopy and Cervical Pathology management guidelines^51^ (ie, cytology if negative for HPV-16 or HPV-18/45; colposcopy if positive for HPV-16 or HPV-18/45) and assisted by a patient navigator in making and keeping their appointment. Before the COVID-19 pause, participants with an unsatisfactory test result were asked to attend clinic-based screening; when accrual resumed, participants with an inadequate result were mailed a second kit.

### Outcomes

The primary outcome was CCS participation within 6 months and defined as return of a mailed self-collection kit or attendance for clinic-based screening. Secondary outcomes were CCS modality (clinic-based or self-collection), CCS test positivity (ie, cytology results requiring colposcopy, positive high-risk HPV test results requiring cytology or colposcopy), and completion of follow-up within 6 months. Exploratory outcomes were histologically confirmed cervical intraepithelial neoplasia grade 2 or higher diagnosis and treatment. Outcomes were assessed by EHR review.

### Sample Size

Calculations were based on a conservative estimate that mailed self-collection would yield a 6% increase in CCS compared to usual care (estimated at 18%).^52^ Sample size was designed to detect the indicated differences in proportions between any 2 groups specifying a 2-sided α = .05 and 80% power and calculated using nQueryAdvisor, version 7.0 (Statsols). Target sample size was 756 participants per group, with a total of 2268 participants in the trial.

### Statistical Analysis

Primary and secondary outcomes were assessed using an intent-to-screen approach (eMethods in Supplement 2). Bivariable tables, Pearson χ^2^ tests, and Kruskal-Wallis nonparametric tests were used to compare demographic and health care characteristics by study group. Log binomial regression was used to calculate screening proportion, participation difference, and relative participation (calculated as relative risk), with corresponding 95% CIs. Overall participation difference and relative participation for SC and SC with patient navigation vs TR were calculated by combining the numerators and denominators from each group. Analyses were conducted with R, version 4.4.1 (R Project for Statistical Computing).

## Results

Between February 20, 2020, and August 31, 2023, there were 18 292 unscreened or underscreened individuals who met the initial inclusion criteria; 3740 were randomly selected for EHR review and attempted telephone contact, and 2577 (68.9%) were reached (Figure). Among those reached, 103 did not meet the additional eligibility criteria, and 2 refused to participate. Of the 2474 individuals randomized (median [IQR] age, 49 [39-57] years), 828 were randomized to TR, 828 to SC, and 818 to SC with patient navigation. There were no statistically significant differences in sociodemographic and health care characteristics across groups (Table 1).

**Figure.**
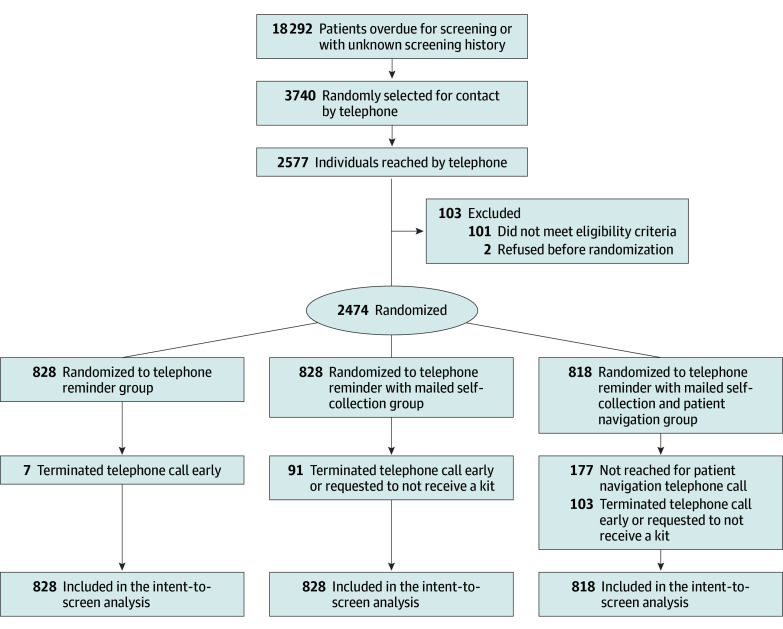
CONSORT Diagram

**Table 1.** Participant Sociodemographic and Health Care Characteristics by Intervention Group

Characteristic	No. (%)			
	All (N = 2474)	TR (n = 828)	TR + SC (n = 828)	TR + SC + patient navigation (n = 818)
Age, median (IQR), y	49 (39-57)	48 (39-57)	49 (39-58)	48 (39-57)
Age at randomization, y				
30-39	653 (26.4)	216 (26.1)	217 (26.2)	220 (26.9)
40-49	653 (26.4)	224 (27.1)	215 (26.0)	214 (26.2)
50-59	725 (29.3)	237 (28.6)	238 (28.7)	250 (30.6)
60-65	443 (17.9)	151 (18.2)	158 (19.1)	134 (16.4)
Race and ethnicity				
Hispanic or Latino	1655 (66.9)	578 (69.8)	548 (66.2)	529 (64.7)
Non-Hispanic Asian	82 (3.3)	27 (3.3)	29 (3.5)	26 (3.2)
Non-Hispanic Black or African American	535 (21.6)	163 (19.7)	173 (20.9)	199 (24.3)
Non-Hispanic White	149 (6.0)	50 (6.0)	57 (6.9)	42 (5.1)
Non-Hispanic other or unknown^a^	53 (2.1)	10 (1.2)	21 (2.5)	22 (2.7)
Primary language				
English	1162 (47.0)	362 (43.7)	389 (47.0)	411 (50.2)
Spanish	1279 (51.7)	451 (54.5)	430 (51.9)	398 (48.7)
Other or unknown	33 (1.3)	15 (1.8)	9 (1.1)	9 (1.1)
Tobacco use				
Current	285 (11.5)	96 (11.6)	85 (10.3)	104 (12.7)
Former	295 (11.9)	104 (12.6)	86 (10.4)	105 (12.8)
Never	1858 (75.1)	610 (73.7)	645 (77.9)	603 (73.7)
Unknown	36 (1.5)	18 (2.2)	12 (1.5)	6 (0.7)
Type of health care coverage				
Private insurance	737 (29.8)	246 (29.7)	246 (29.7)	245 (30.0)
Publicly funded financial assistance plan	1388 (56.1)	476 (57.5)	461 (55.7)	451 (55.1)
Medicaid	203 (8.2)	60 (7.3)	69 (8.3)	74 (9.0)
Medicare	80 (3.2)	27 (3.3)	30 (3.6)	23 (2.8)
Other	66 (2.7)	19 (2.3)	22 (2.7)	25 (3.1)
Time overdue for screening, y				
<5	269 (10.9)	98 (11.8)	89 (10.8)	82 (10.0)
5-10	1593 (64.4)	523 (63.2)	526 (63.5)	544 (66.5)
>10	417 (16.9)	131 (15.8)	147 (17.8)	139 (17.0)
No prior screen record	195 (7.9)	76 (9.2)	66 (8.0)	53 (6.5)
Time since last screening among those with prior screen record, median (IQR), y	7.22 (5.92-9.09)	7.23 (5.87-9.09)	7.23 (5.99-9.29)	7.19 (5.92-8.96)
Have designated primary care clinician	1484 (60.0)	480 (58.0)	500 (60.4)	504 (61.6)
No. of ambulatory care encounters in past 5 y, median (IQR)^b^	16 (7-38)	14 (7-34)	17 (7-42)	17 (7-39)
Randomization year				
2020	522 (21.1)	175 (21.1)	175 (21.1)	172 (21.0)
2021	916 (37.0)	305 (36.8)	305 (36.8)	306 (37.4)
2022	748 (30.2)	250 (30.2)	252 (30.4)	246 (30.1)
2023	288 (11.6)	98 (11.8)	96 (11.6)	94 (11.5)

Abbreviations: SC, self-collection; TR, telephone reminder.

^a^ The non-Hispanic other or unknown category includes American Indian or Alaska Native and Native Hawaiian or Other Pacific Islander, which were grouped together owing to small sample sizes.

^b^ Number of ambulatory care encounters included any documented non–emergency department outpatient encounter, including laboratory testing, pharmacy, office visits, and outpatient procedures. Variable excludes participants who refused the assigned intervention after randomization (n = 2273).

Across the interventions, 7 individuals (0.9%) refused TR, 91 (11.0%) refused SC, and 103 (12.6%) refused SC with patient navigation (Figure). Among participants randomized to SC with patient navigation and mailed a kit, 157 of 715 (22.0%) returned the kit before the patient navigation telephone call and 177 of 558 (31.7%) were not reached.

A total of 144 individuals (17.4%) participated in TR, 340 (41.1%) participated in SC, and 381 (46.6%) participated in SC with patient navigation (eFigure in Supplement 2). Comparing SC to TR, participation difference was 23.7% (95% CI, 19.4%-27.9%), and relative participation was 2.36 (95% CI, 1.99-2.80) times higher (Table 2). Comparing SC with patient navigation to TR, participation difference was 29.2% (95% CI, 24.9%-33.5%) and relative participation was 2.68 (95% CI, 2.27-3.16) times higher. Overall, participants who were mailed self-collection kits (SC and SC with patient navigation) had 43.8% screening participation; participation difference and relative participation compared to TR were 26.4% (95% CI, 22.9%-29.9%) and 2.52 (95% CI, 2.15-2.95) time higher, respectively. Comparing SC with patient navigation to SC, screening difference was 5.5% (95% CI, 0.7%-10.3%) and relative participation was 1.13 (95% CI, 1.02-1.27) times higher.

**Table 2.** Screening Participation by Study Group

Randomization group	No. randomized	Screening participation, No. (%)		Overall screening participation (95% CI)^a^					
				Compared with telephone reminder		Telephone reminder with mailed self-collection and patient navigation vs telephone reminder with mailed self-collection		Overall telephone reminder with mailed self-collection vs telephone reminder	
		Clinic based + self-collection	Self-collection only	Relative participation	Participation difference, %	Relative participation	Participation difference, %	Relative participation	Participation difference, %
Telephone reminder	828	144 (17.4)	NA	1 [Reference]	0 [Reference]	NA	NA	1 [Reference]	0 [Reference]
Telephone reminder with mailed self-collection	828	340 (41.1)	283 (34.1)	2.36 (1.99-2.80)	23.7 (19.4-27.9)	1 [Reference]	0 [Reference]	2.52 (2.15-2.95)	26.4 (22.9-29.9)
Telephone reminder with mailed self-collection and patient navigation	818	381 (46.6)	326 (39.9)	2.68 (2.27-3.16)	29.2 (24.9-33.5)	1.13 (1.02-1.27)	5.5 (0.7-10.3)		

Abbreviation: NA, not applicable.

^a^ 95% CIs calculated using normal approximation.

CCS participation by age, race and ethnicity, and time overdue for screening are summarized in Table 3. CCS participation was greater in SC and SC with patient navigation compared to TR across all strata, with notable differences between SC and SC with patient navigation among participants aged 50 to 59 years, those of non-Hispanic Black and non-Hispanic other or unknown race and ethnicity, and those with no prior CCS record.

**Table 3.** Overall Participation in Cervical Cancer Screening, Stratified by Age, Race and Ethnicity, and Time Since Last Screening Test

Variable	No./total No. (%)		
	Telephone reminder (n = 828)	Telephone reminder with mailed self-collection (n = 828)	Telephone reminder with mailed self-collection and patient navigation (n = 818)
Age at randomization, y			
30-39	40/216 (18.5)	77/217 (35.5)	84/220 (38.2)
40-49	41/224 (18.3)	88/215 (40.9)	103/214 (48.1)
50-59	47/237 (19.8)	100/238 (42.0)	130/250 (52.0)
60-65	16/151 (10.6)	75/158 (47.5)	64/134 (47.8)
Race and ethnicity			
Hispanic or Latino	109/578 (18.9)	240/548 (43.8)	255/529 (48.2)
Non-Hispanic Asian	3/27 (11.1)	11/29 (37.9)	9/26 (34.6)
Non-Hispanic Black or African American	24/163 (14.7)	61/173 (35.3)	93/199 (46.7)
Non-Hispanic White	8/50 (16.0)	20/57 (35.1)	18/42 (42.9)
Non-Hispanic other or unknown^a^	0	8/21 (38.1)	6/22 (27.3)
Time overdue for screening, y			
<5	15/98 (15.3)	41/89 (46.1)	40/82 (48.8)
5-10	103/523 (19.7)	223/526 (42.4)	256/544 (47.1)
>10	14/131 (10.7)	51/147 (34.7)	60/139 (43.2)
No prior screen record	12/76 (15.8)	25/66 (37.9)	25/53 (47.2)

^a^ The non-Hispanic other or unknown category includes American Indian or Alaska Native and Native Hawaiian or Other Pacific Islander, which were grouped together owing to small sample sizes.

Among participants who were mailed a self-collection kit (SC and SC with patient navigation) and participated in screening, 609 of 721 (84.6%) did so by returning a self-collection kit (eFigure in Supplement 2). Among participants who returned a self-collection kit, 491 (80.6%) had a negative high-risk HPV test result; 7 (1.2%) had positive HPV-16 or HPV-18/45 test results; 77 (12.6%) had positive test results for other high-risk HPV types; and 34 (5.6%) had a test that was unsatisfactory for testing. All participants with a positive and most with a negative or unsatisfactory test result were notified of results.

Overall, 84 of 609 participants (13.8%) had a positive high-risk HPV test result that required follow-up (77 with cytology and 7 with colposcopy). The proportion who completed follow-up was 22 of 36 (61.1%) for SC and 33 of 48 (68.8%) for SC with patient navigation. Among those who attended clinic-based screening, 9 of 256 (3.5%) had a positive test result and 4 of 9 (44.4%) completed follow-up. Three participants who returned a self-collection kit and none who attended clinic-based screening were diagnosed with cervical intraepithelial neoplasia grade 2 or higher.

One participant in the SC with patient navigation group reported an expected adverse event (mild discomfort). No unexpected adverse events were reported.

## Discussion

This pragmatic randomized clinical trial is, to our knowledge, the first to evaluate self-collection in a US public safety-net health system. The results demonstrate that mailing self-collection kits to the homes of underscreened individuals following TR is effective for increasing CCS participation in safety-net settings. Additional, albeit modest, increases were attained by pairing SC with patient navigation. Looking collectively at the SC and SC with patient navigation groups, CCS participation was approximately 44% among participants mailed a self-collection kit, a relative participation rate 2.5 times higher than with TR. Moreover, among participants provided this option, more than 80% of those screened did so by returning a self-collection kit, providing further evidence for the preference and improved suitability of this approach within the safety-net patient population.

The relative increase in CCS participation with mailed self-collection kits in the PRESTIS trial is consistent with the relative participation rates of previous trials. In a recent meta-analysis of 28 trials, Costa et al^9^ reported a 2.5-fold relative participation in screening with mailed self-collection vs usual care. Notably distinct from previous trials are the large screening proportions attained in the SC (41.1%) and SC with patient navigation (46.6%) groups, as compared to the global estimate of 24% reported in the meta-analysis. Similarly, the absolute difference in screening participation in this trial (26% for SC and SC with navigation combined) is double that of the global estimate of 13%.^9^ The higher participation proportion and difference may be attributed in part to the setting in which it was conducted. Previous mailed self-collection trials have predominantly been conducted in high-resource countries with organized CCS programs and universal health care coverage.^53^ Few studies have been completed in the US^34,35^ and only 1 in an underserved population.^36^ The higher use of self-collection kits in the PRESTIS trial is likely reflective of the considerable structural barriers to CCS experienced by the underserved and predominantly racially and ethnically minoritized populations who receive care in safety-net settings. In a nested survey within the PRESTIS trial, we identified that more than 80% of underscreened participants had an annual household income below the federal poverty level,^54^ and limited access to health care was among the top barriers to clinic-based screening.^43,53^

Another factor that likely contributed to high participation in SC is the trial’s pairing of SC with TR. While the requirement to reach individuals for TR reduced the pool of potential participants by more than 30%, this was done to ensure that participants currently lived in the county (otherwise ineligible for health system services) and that their mailing address could be verified and/or updated given the high degree of transient residence in the patient population. Participants across all groups received TR from a bilingual patient navigator and were provided information on the importance of CCS. This may have prompted participants to use the kit when it arrived in the mail, potentially more so than the use of an invitation letter.^9^ Conversely, the increased participation with SC with patient navigation vs SC was small (46.6% vs 41.1%), suggesting that the education and/or encouragement provided during the first telephone encounter may have been sufficient to promote self-collection among most kit users, with only some benefitting from additional intervention. Furthermore, less than 70% of participants in the SC with patient navigation group could be reached for the patient navigation telephone call, further bringing into question the cost-effectiveness of this approach. Future research will be important to determine the incremental value of this approach relative to other interventions. Future trials would benefit from evaluating whether similar improvements could be made with lower-cost interventions such as automated reminders and text messaging.

### Limitations

The trial’s findings should be interpreted within the context of its limitations. First, while care was given to maximize the pragmaticism of the trial, participants were made aware that self-collection was being offered as part of a research study, which may have influenced participation. For a period of the trial, access to in-person health services was limited due to precautions around the COVID-19 pandemic, which could have inflated relative participation in self-collection vs clinic-based screening. However, only 20% of the data were collected in 2020 before full resumption of clinical services, and year-year comparisons do not indicate temporal differences by screening modality. Interventions across all arms were delivered by the same patient navigators, who were not blinded to the assignment of participants. However, SC with patient navigation was designed to be different from SC in terms of the dose of patient navigation interactions rather than content, minimizing potential contamination across SC and SC with patient navigation. Refusal of the intervention was considerably higher in SC and SC with patient navigation compared to TR given that participants in the SC and SC with patient navigation groups could request to not receive a kit in addition to terminating the telephone call early. Reasons for refusal were not systematically documented, but free-text entries indicate that refusals were commonly due to unspecified reasons and to participants declining to verify and/or update their mailing address (potentially indicating a degree of distrust with the health system and/or fear of disclosing their place of residence). Specific barriers addressed by patient navigators were also not systematically documented; however, qualitative analyses of participants’ experiences with patient navigation are underway.

Additional limitations include the trial not being powered to test differences in follow-up or to evaluate attendance to specific follow-up modalities. This is pertinent because individuals with positive high-risk HPV test results must undergo triage with cytology or be referred directly to colposcopy; screening is not considered complete without cytology triage for those in this pathway.^54^ With the anticipated FDA approval of home-based self-collection, future trials should evaluate attendance to guideline-concordant follow-up. Self-collected samples were tested using the Aptima HPV test, as this was the test that could be performed internally using the available infrastructure of the health system laboratory. However, this is a messenger RNA test with lower sensitivity for detecting high-risk HPV on self-collected samples than DNA tests^11^ and is not currently under consideration for FDA approval with self-collection. Finally, self-collection was FDA approved for use in clinical settings following completion of the trial. As self-collection becomes integrated in clinical workflows, it will be important to determine how mailed self-collection should be implemented alongside clinic-based self-collection.

## Conclusions

The PRESTIS randomized clinical trial has implications for clinical practice and policy in the US. The increased CCS participation in SC over TR underscores the value of implementing this alternative approach to CCS, particularly in safety-net health settings and among underserved populations who face substantial structural barriers to clinic-based CCS. With the anticipated FDA approval of home-based self-collection, these findings, along with forthcoming cost-effectiveness data from the PRESTIS trial, could inform practices and resource allocation decisions in both safety-net and broader health care settings.

## Notes

Supplement 1.
Trial Protocol

Supplement 2. **eMethods.** Statistical Analysis Plan **eFigure.** Study outcomes: primary (screening participation), secondary (screening test results and attendance for clinical follow-up), and exploratory (diagnosis and treatment)

Supplement 3.
Data Sharing Statement

## References

[1] Global strategy to accelerate the elimination of cervical cancer as a public health problem. World Health Organization. November 17, 2020. Accessed September 2, 2024. https://www.who.int/publications/i/item/9789240014107

[2] Burger EA, Smith MA, Killen J, . Projected time to elimination of cervical cancer in the USA: a comparative modelling study. Lancet Public Health. 2020;5(4):e213-e222. doi:10.1016/S2468-2667(20)30006-2 32057315 PMC8715100

[3] Sabatino SA, Thompson TD, White MC, . Up-to-date breast, cervical, and colorectal cancer screening test use in the United States, 2021. Prev Chronic Dis. 2023;20:E94. doi:10.5888/pcd20.230071 37884318 PMC10625435

[4] Healthy People 2030: objectives and data. US Department of Health and Human Services. Accessed May 22, 2025. https://odphp.health.gov/healthypeople/objectives-and-data/browse-objectives/cancer

[5] Cancer Trends Progress Report: cervical cancer screening. National Cancer Institute. Accessed July 20, 2024. https://progressreport.cancer.gov/detection/cervical_cancer

[6] Suk R, Hong YR, Rajan SS, Xie Z, Zhu Y, Spencer JC. Assessment of US Preventive Services Task Force guideline-concordant cervical cancer screening rates and reasons for underscreening by age, race and ethnicity, sexual orientation, rurality, and insurance, 2005 to 2019. JAMA Netw Open. 2022;5(1):e2143582. doi:10.1001/jamanetworkopen.2021.43582 35040970 PMC8767443

[7] Burger EA, Jansen EEL, de Bondt D, . Disparities in cervical cancer elimination timeframes in the United States: a comparative modeling study. J Natl Cancer Inst. 2025;djae319. doi:10.1093/jnci/djae319 39798139 PMC12229461

[8] Curry SJ, Krist AH, Owens DK, ; US Preventive Services Task Force. Screening for cervical cancer: US Preventive Services Task Force recommendation statement. JAMA. 2018;320(7):674-686. doi:10.1001/jama.2018.10897 30140884

[9] Costa S, Verberckmoes B, Castle PE, Arbyn M. Offering HPV self-sampling kits: an updated meta-analysis of the effectiveness of strategies to increase participation in cervical cancer screening. Br J Cancer. 2023;128(5):805-813. doi:10.1038/s41416-022-02094-w 36517552 PMC9977737

[10] Arbyn M, Smith SB, Temin S, Sultana F, Castle P; Collaboration on Self-Sampling and HPV Testing. Detecting cervical precancer and reaching underscreened women by using HPV testing on self samples: updated meta-analyses. BMJ. 2018;363:k4823. doi:10.1136/bmj.k4823 30518635 PMC6278587

[11] Arbyn M, Castle PE, Schiffman M, Wentzensen N, Heckman-Stoddard B, Sahasrabuddhe VV. Meta-analysis of agreement/concordance statistics in studies comparing self- vs clinician-collected samples for HPV testing in cervical cancer screening. Int J Cancer. 2022;151(2):308-312. doi:10.1002/ijc.33967 35179777

[12] Nelson EJ, Maynard BR, Loux T, Fatla J, Gordon R, Arnold LD. The acceptability of self-sampled screening for HPV DNA: a systematic review and meta-analysis. Sex Transm Infect. 2017;93(1):56-61. doi:10.1136/sextrans-2016-052609 28100761

[13] Reynolds S. FDA approves HPV tests that allow for self-collection in a health care setting. News release. National Cancer Institute. July 24, 2024. Accessed May 22, 2025. https://prevention.cancer.gov/news-and-events/news/fda-approves-hpv-tests-allow-self-collection-health-care-setting

[14] NCI cervical cancer ‘last mile’ initiative. National Cancer Institute. Accessed November 6, 2024.https://prevention.cancer.gov/research-areas/networks-consortia-programs/last-mile

[15] Inturrisi F, Aitken CA, Melchers WJG, . Clinical performance of high-risk HPV testing on self-samples versus clinician samples in routine primary HPV screening in the Netherlands: An observational study. Lancet Reg Health Eur. 2021;11:100235. doi:10.1016/j.lanepe.2021.100235 34918001 PMC8642706

[16] Sultana F, Gertig DM, English DR, . HPV self-sampling and follow-up over two rounds of cervical screening in Australia—the iPap trial. J Med Screen. 2022;29(3):185-193. doi:10.1177/09691413221080635 35313763

[17] Bais AG, van Kemenade FJ, Berkhof J, . Human papillomavirus testing on self-sampled cervicovaginal brushes: an effective alternative to protect nonresponders in cervical screening programs. Int J Cancer. 2007;120(7):1505-1510. doi:10.1002/ijc.22484 17205514

[18] Gök M, Heideman DA, van Kemenade FJ, . HPV testing on self collected cervicovaginal lavage specimens as screening method for women who do not attend cervical screening: cohort study. BMJ. 2010;340:c1040. doi:10.1136/bmj.c1040 20223872 PMC2837143

[19] Giorgi Rossi P, Marsili LM, Camilloni L, ; Self-Sampling Study Working Group. The effect of self-sampled HPV testing on participation to cervical cancer screening in Italy: a randomised controlled trial (ISRCTN96071600). Br J Cancer. 2011;104(2):248-254. doi:10.1038/sj.bjc.6606040 21179038 PMC3031894

[20] Szarewski A, Cadman L, Mesher D, . HPV self-sampling as an alternative strategy in non-attenders for cervical screening—a randomised controlled trial. Br J Cancer. 2011;104(6):915-920. doi:10.1038/bjc.2011.48 21343937 PMC3065284

[21] Virtanen A, Nieminen P, Niironen M, Luostarinen T, Anttila A. Self-sampling experiences among non-attendees to cervical screening. Gynecol Oncol. 2014;135(3):487-494. doi:10.1016/j.ygyno.2014.09.019 25284037

[22] Wikström I, Lindell M, Sanner K, Wilander E. Self-sampling and HPV testing or ordinary Pap-smear in women not regularly attending screening: a randomised study. Br J Cancer. 2011;105(3):337-339. doi:10.1038/bjc.2011.236 21730977 PMC3172898

[23] Sancho-Garnier H, Tamalet C, Halfon P, . HPV self-sampling or the Pap-smear: a randomized study among cervical screening nonattenders from lower socioeconomic groups in France. Int J Cancer. 2013;133(11):2681-2687. doi:10.1002/ijc.28283 23712523

[24] Haguenoer K, Sengchanh S, Gaudy-Graffin C, . Vaginal self-sampling is a cost-effective way to increase participation in a cervical cancer screening programme: a randomised trial. Br J Cancer. 2014;111(11):2187-2196. doi:10.1038/bjc.2014.510 25247320 PMC4260034

[25] Giorgi Rossi P, Fortunato C, Barbarino P, ; HPV Self-sampling Italian Working Group. Self-sampling to increase participation in cervical cancer screening: an RCT comparing home mailing, distribution in pharmacies, and recall letter. Br J Cancer. 2015;112(4):667-675. doi:10.1038/bjc.2015.11 25633037 PMC4333501

[26] Racey CS, Gesink DC, Burchell AN, Trivers S, Wong T, Rebbapragada A. Randomized intervention of self-collected sampling for human papillomavirus testing in under-screened rural women: uptake of screening and acceptability. J Womens Health (Larchmt). 2016;25(5):489-497. doi:10.1089/jwh.2015.5348 26598955

[27] Sultana F, English DR, Simpson JA, . Home-based HPV self-sampling improves participation by never-screened and under-screened women: results from a large randomized trial (iPap) in Australia. Int J Cancer. 2016;139(2):281-290. doi:10.1002/ijc.30031 26850941

[28] Ivanus U, Jerman T, Fokter AR, . Randomised trial of HPV self-sampling among non-attenders in the Slovenian cervical screening programme ZORA: comparing three different screening approaches. Radiol Oncol. 2018;52(4):399-412. doi:10.2478/raon-2018-0036 30216191 PMC6287183

[29] Tranberg M, Bech BH, Blaakær J, Jensen JS, Svanholm H, Andersen B. HPV self-sampling in cervical cancer screening: the effect of different invitation strategies in various socioeconomic groups—a randomized controlled trial. Clin Epidemiol. 2018;10:1027-1036. doi:10.2147/CLEP.S164826 30197540 PMC6112594

[30] Elfström KM, Sundström K, Andersson S, . Increasing participation in cervical screening by targeting long-term nonattenders: randomized health services study. Int J Cancer. 2019;145(11):3033-3039. doi:10.1002/ijc.32374 31032904

[31] Jalili F, O’Conaill C, Templeton K, . Assessing the impact of mailing self-sampling kits for human papillomavirus testing to unscreened non-responder women in Manitoba. Curr Oncol. 2019;26(3):167-172. doi:10.3747/co.26.4575 31285661 PMC6588079

[32] Lilliecreutz C, Karlsson H, Spetz Holm AC. Participation in interventions and recommended follow-up for non-attendees in cervical cancer screening -taking the women’s own preferred test method into account—a Swedish randomised controlled trial. PLoS One. 2020;15(7):e0235202. doi:10.1371/journal.pone.0235202 32614875 PMC7332065

[33] Darlin L, Borgfeldt C, Forslund O, . Comparison of use of vaginal HPV self-sampling and offering flexible appointments as strategies to reach long-term non-attending women in organized cervical screening. J Clin Virol. 2013;58(1):155-160. doi:10.1016/j.jcv.2013.06.029 23867008

[34] Winer RL, Lin J, Anderson ML, . Strategies to increase cervical cancer screening with mailed human papillomavirus self-sampling kits: a randomized clinical trial. JAMA. 2023;330(20):1971-1981. doi:10.1001/jama.2023.21471 38015219 PMC10685881

[35] Winer RL, Lin J, Tiro JA, . Effect of mailed human papillomavirus test kits vs usual care reminders on cervical cancer screening uptake, precancer detection, and treatment: a randomized clinical trial. JAMA Netw Open. 2019;2(11):e1914729. doi:10.1001/jamanetworkopen.2019.14729 31693128 PMC6865279

[36] Reiter PL, Shoben AB, Cooper S, . A mail-based HPV self-collection program to increase cervical cancer screening in Appalachia: results of a group randomized trial. Cancer Epidemiol Biomarkers Prev. 2025;34(1):159-165. doi:10.1158/1055-9965.EPI-24-0999 39445831 PMC11717618

[37] Amboree TL, Damgacioglu H, Sonawane K, Adsul P, Montealegre JR, Deshmukh AA. Recent trends in cervical cancer incidence, stage at diagnosis, and mortality according to county-level income in the United States, 2000-2019. Int J Cancer. 2024;154(9):1549-1555. doi:10.1002/ijc.34860 38270521 PMC11410343

[38] Chokshi DA, Chang JE, Wilson RM. Health reform and the changing safety net in the United States. N Engl J Med. 2016;375(18):1790-1796. doi:10.1056/NEJMhpr1608578 27806232

[39] Montealegre JR, Anderson ML, Hilsenbeck SG, . Mailed self-sample HPV testing kits to improve cervical cancer screening in a safety net health system: protocol for a hybrid effectiveness-implementation randomized controlled trial. Trials. 2020;21(1):872. doi:10.1186/s13063-020-04790-5 33087164 PMC7580009

[40] Impact of the Health Center Program. Health Resources and Services Administration. Accessed September 3, 2024. https://bphc.hrsa.gov/about-health-center-program/impact-health-center-program

[41] Cohen CM, Wentzensen N, Castle PE, . Racial and ethnic disparities in cervical cancer incidence, survival, and mortality by histologic subtype. J Clin Oncol. 2023;41(5):1059-1068. doi:10.1200/JCO.22.01424 36455190 PMC9928618

[42] Amboree TL, Damgacioglu H, Sonawane K, Adsul P, Montealegre JR, Deshmukh AA. Recent trends in cervical cancer incidence, stage at diagnosis, and mortality according to county-level income in the United States, 2000-2019. Int J Cancer. 2024;154(9):1549-1555. doi:10.1002/ijc.34860 38270521 PMC11410343

[43] Amboree TL, Montealegre JR, Parker SL, . National breast, cervical, and colorectal cancer screening use in federally qualified health centers. JAMA Intern Med. 2024;184(6):671-679. doi:10.1001/jamainternmed.2024.0693 38683574 PMC11059050

[44] Parker S, Deshmukh AA, Chen B, . Perceived barriers to cervical cancer screening and motivators for at-home human papillomavirus self-sampling during the COVID-19 pandemic: results from a telephone survey. Elife. 2023;12:e84664. doi:10.7554/eLife.84664 37232493 PMC10335828

[45] Ogunwale AN, Sangi-Haghpeykar H, Montealegre J, Cui Y, Jibaja-Weiss M, Anderson ML. Non-utilization of the Pap test among women with frequent health system contact. J Immigr Minor Health. 2016;18(6):1404-1412. doi:10.1007/s10903-015-0287-9 26424729

[46] Amboree TL, Parker SL, Bulsara S, . Cervical cancer screening among English- and Spanish-speaking Hispanic women in an urban safety net health system, 2015-2020. BMC Womens Health. 2023;23(1):309. doi:10.1186/s12905-023-02448-3 37316815 PMC10268437

[47] Freeman HP. Patient navigation: a community based strategy to reduce cancer disparities. J Urban Health. 2006;83(2):139-141. doi:10.1007/s11524-006-9030-0 16736361 PMC2527166

[48] Paskett ED, Harrop JP, Wells KJ. Patient navigation: an update on the state of the science. CA Cancer J Clin. 2011;61(4):237-249. doi:10.3322/caac.20111 21659419 PMC3623288

[49] Harris PA, Taylor R, Thielke R, Payne J, Gonzalez N, Conde JG. Research electronic data capture (REDCap)—a metadata-driven methodology and workflow process for providing translational research informatics support. J Biomed Inform. 2009;42(2):377-381. doi:10.1016/j.jbi.2008.08.010 18929686 PMC2700030

[50] Harris PA, Taylor R, Minor BL, ; REDCap Consortium. The REDCap consortium: building an international community of software platform partners. J Biomed Inform. 2019;95:103208. doi:10.1016/j.jbi.2019.103208 31078660 PMC7254481

[51] Perkins RB, Guido RS, Castle PE, ; 2019 ASCCP Risk-Based Management Consensus Guidelines Committee. 2019 ASCCP risk-based management consensus guidelines for abnormal cervical cancer screening tests and cancer precursors. J Low Genit Tract Dis. 2020;24(2):102-131. doi:10.1097/LGT.0000000000000525 32243307 PMC7147428

[52] Massad LS, Einstein MH, Huh WK, ; 2012 ASCCP Consensus Guidelines Conference. 2012 updated consensus guidelines for the management of abnormal cervical cancer screening tests and cancer precursors. Obstet Gynecol. 2013;121(4):829-846. doi:10.1097/AOG.0b013e3182883a34 23635684

[53] Waheed DE, Burdier FR, Eklund C, . An update on one-dose HPV vaccine studies, immunobridging and humoral immune responses—a meeting report. Prev Med Rep. 2023;35:102368. doi:10.1016/j.pmedr.2023.102368 37680853 PMC10480621

[54] Parker SL, Amboree TL, Bulsara S, . Self-sampling for human papillomavirus testing: acceptability in a U.S. safety net health system. Am J Prev Med. 2024;66(3):540-547. doi:10.1016/j.amepre.2023.10.020 37935320 PMC12177980

