# Supplementary material for: Self-Collection for Cervical Cancer Screening in a Safety-Net Setting: The PRESTIS Randomized Clinical Trial
Source: JAMA Intern Med. 2025 Jun 6;185(9):1119–27. doi: 10.1001/jamainternmed.2025.2971 (PMC12144659; doi:10.1001/jamainternmed.2025.2971)
Supplement: Supplement 1. — Trial Protocol [file jamainternmed-e252971-s001.pdf]

Institutional Review Board Protocol: A Randomized Controlled Trial of Mailed Self-Sample HPV Testing to Increase Cervical Cancer Screening Participation among Minority/Underserved Women in an Integrated Safety Net Healthcare System

Background Information

The implementation of clinic-based Papanicolaou (Pap) screening for cervical cancer has dramatically reduced the incidence of this disease in the US and other countries with widespread screening programs. However, despite over $5.4 billion spent annually on routine screening, almost 20% of US women remain at high risk for cervical cancer due to screening non-attendance (i.e., their inability or unwillingness to periodically attend for clinic-based screening according to the screening guidelines). In fact, over half of the 13,000 cases of invasive cervical cancer diagnosed in the US each year are among screening non-attendees. Cervical cancer results in over 4,000 deaths annually, and its treatment and follow-up costs total over $440 million. While screening non-attendance is largely due to inadequate access to preventive care, multiple personal and cultural barriers also affect women, participation in timely screening. These barriers include language and cultural differences with providers, discomfort during a pelvic exam, education/literacy, and health beliefs. Our research suggests that modesty concerns and the unacceptability of a male physician may also play a role. Many of these factors continue to adversely impact screening participation after medically underserved women gain access to healthcare. While the availability of licensed vaccines to prevent infection with the etiologic agent, high- risk human papillomavirus (HR-HPV), creates new opportunities to reduce the incidence of both cervical cancer and pre-invasive cervical disease, low rates of vaccine uptake in the US means that cervical cancer risk will persist for at least several generations of women. Cost-effective strategies to improve existing screening programs thus will be required for the foreseeable future.

At present, evidence-based, client-directed strategies such as patient reminders and recalls, one-on-one patient education, and patient navigation, are the basis for behavioral interventions to increase cervical cancer screening rates. Patient reminders inform patients that they are due for screening, while recalls inform them they are past-due. Patient navigation, a barrier-focused intervention, involves individual interactions with patients to address individual-level barriers along the continuum of care through to a specific endpoint (e.g., cancer screening). These existing client-directed strategies alone are often unable to resolve many of the barriers faced by screening non-attendees. Of note, we recently found that as many as 10% of women attending safety net community health centers in Harris County reported never having had a Pap test despite having visited those clinics an average of nearly four times in the past year. This observation suggests that current standard of care cervical cancer screening may be simply unacceptable or unfeasible for many women.

Testing self-collected cervicovaginal samples for HR-HPV may be an effective strategy to overcome the multiple barriers that hinder clinic-based screening. Testing for HR-HPV has been shown to be equally or more sensitive than liquid-based cytology for detecting invasive cervical cancer and high-grade lesions. Reflecting its sensitivity, HR-HPV testing using provider-collected samples is now recommended by the US Food and Drug Administration (FDA) as an alternative to Pap test screening. Recently, it has become the primary screening tool in The Netherlands and Australia and is being considered as such in several other countries. Substantial evidence indicates that samples can be collected by providers or by women themselves, with similar sensitivity for detecting HR-HPV nucleic acid. Numerous studies, including our own, have demonstrated high acceptability for a self-sampling modality. In our own research, a sizeable proportion of women indicated a preference to utilize self-collected samples rather than undergo a pelvic examination by a healthcare provider

and considered self-sampling more convenient and less stressful than clinic-based screening. These and other data thus pave an exciting path for the expansion of primary screening from clinical settings into women's homes through mailed self-sample HPV testing kits.

Mailed self-sample HPV testing has been evaluated in multiple trials in Europe, Australia, and Canada as an additive strategy to increase primary screening participation among screening non-attendees of organized population-based screening programs. These trials have reported that as many as 10-39% of non-attendees complete and return the mailed self-sampling kits. Despite these promising results, there has only been one published trial in the US to evaluate mailed self-sample HPV testing in the analogous setting of an integrated health system, i.e., a health system that focuses on coordinated care provision across the care continuum. The HOME (Home-based Options to Make Cervical Cancer Screening Easy) trial is a pragmatic randomized controlled trial (RCT) that is being conducted within the Kaiser Permanente system, a highly organized, private health system that serves a predominantly non-minority and privately insured patient population. To date, no RCTs have been conducted in safety net healthcare systems nor in a predominantly racially/ethnically minority patient population. Evaluating mailed self-sample HPV testing in this context is important both because safety net health systems (i.e., those that offer access to care regardless of the patient's ability to pay) serve a large proportion of socioeconomically disadvantaged individuals in the US and because racial/ethnic minorities, particularly Hispanic and non-Hispanic black women, carry a disproportionate burden of cervical disease.

The PRESTIS Trial (Prospective Evaluation of Self-Testing to Increase Screening) described here is a parallel, single-blinded, three-arm RCT comparing the effectiveness of three outreach strategies for improving cervical cancer screening participation and attendance for clinical follow-up among women age 30-65 years who are overdue for Pap test screening. Being overdue

for a Pap test is defined as not having a Pap test in the past 3.5 years or more, which is based on a three year screening interval and a 6- month grace period. The study arms are: 1) telephone recall (control arm); 2) telephone recall with mailed self-sample HPV testing kit (intervention arm); and (3) telephone recall with mailed self- sample HPV testing kit and patient navigation (intervention plus arm). The primary outcome is completion of primary screening, defined as completion and return of mailed self- sample kit or completion of a clinic-based Pap test. Secondary outcomes are predictors of screening and attendance for clinical follow-up among women with a positive screening test. Additional exploratory outcomes are detection and treatment of cervical precancers and cost-effectiveness. The contribution of the PRESTIS Trial is significant because it will define how and under what conditions self-sample HPV testing can be used cost-effectively by safety net health systems for primary screening and early detection of cervical precancer among non-attendees.

Background for administrative supplement to expand enrollment to Asian/Asian American women: The patient population of the safety net health system (serving primarily low income women) where the PRESTIS trial is embedded is 67% Hispanic, 22% non-Hispanic Black, 7% non-Hispanic White, 5% Asian, and 2% other race/ethnicity. Reflecting this demographic composition and logistical constraints of implementing the trial in multiple languages, eligibility is currently limited to patients who speak English or Spanish.

Asian/Asian American women who speak English currently comprise a small proportion of trial participants (4%). Furthermore, Asian/Asian American women who speak languages other than English (particularly Vietnamese, the primary language of 68% of Asian patients) are ineligible for the trial. The small number of Asian/Asian American patients in the trial is problematic as it precludes their inclusion in subgroup analyses and limits the generalizability of trial findings.

The inclusion of Asian/Asian American women in self-sampling trials is particularly important given that cervical cancer screening participation is lowest among Asian/Asian American women in the U.S. (63.7% vs. 79.5% among non-Hispanic Whites (7), and certain Asian subpopulations, notably Vietnamese women, have higher cervical cancer incidence compared to women of other race/ethnicities (8, 9). With the administrative supplement, we will expand the PRESTIS trial by adapting patient education materials and navigation strategies to recruit Asian women who are not up-to-date on their cervical cancer screening.

Purpose and Objectives

The overall purpose of this protocol is to evaluate the effectiveness of self-sample HPV testing with patient navigation to increase cervical cancer screening among women who otherwise do not regularly attend for clinic-based screening (i.e., Pap test).

The specific aims of this protocol are to: SPECIFIC AIM 1: Compare the effectiveness of mailed self-sample HPV testing alone and in combination with patient navigation to increase primary screening participation (primary outcome) and clinical follow-up (secondary outcome). Hypothesis 1.1: Primary screening participation and clinical follow-up will be significantly greater among women randomized to receive telephone recall with mailed self-sampling kits versus telephone recall alone. Hypothesis 1.2: There will be significant incremental gains in screening participation and clinical follow-up among women randomized to receive mailed self-sampling kits in combination with patient navigation.

Analytical sub-aims of the protocol are to: 1) Describe characteristics of women who under-attend for cervical cancer screening in a safety net health system; 2) Explore patterns of detection and

treatment of cervical pre-cancers across study arms; 3) Compare characteristics of screening participants across study arms.

We will also conduct a nested study within the randomized controlled trial to assess acceptability of and experiences with self-sample HPV testing among underscreened women in our safety net health system. The nested study has two components: a telephone survey and in-depth qualitative interviews. In the remainder of the protocol these will be referred to as "Telephone Survey" and "Qualitative Interviews."

Because of the COVID-19 pandemic, we have revised some elements of the trial, as reflected throughout the protocol. One of these revisions includes expansion of analytic sub-aim 1 (above) to include monitoring of ambulatory, and more specifically, primary care utilization in addition to cervical cancer screening. The COVID-19 era sub-aims are: 1) To compare changes in cervical cancer screening and primary care utilization (overall and by face-to-face vs. telehealth modalities) among socioeconomic, racial/ethnic, and nativity subgroups in the 12-months following the COVID-19 emergency declaration; and 2) To compare the role of individual and neighborhood-level factors on cervical cancer screening and primary care utilization (overall and modality-specific) before and after the COVID-19 emergency declaration. In the remainder to the protocol, we will refer to COVID-19 era analytic sub- aims.

After receiving an administrative supplement, we have revised the trial to include Asian/Asian American women who are currently under-presented in the trial and in the broader literature on self-sampling. In doing so, we will ensure that the trial is powered to rigorously assess and compare screening participation and other outcomes across racial/ethnic and linguistic

subpopulations inclusive of Asian/Asian American women. Collectively, the data generated in pursuit of these aims and those of the larger trial will define the impact of mailed self-sample HPV testing among medically underserved Asian/Asian American and other racial/ethnic minority women in safety net health system settings.

Protocol Risks/Subjects

Risk Category: Category 1: Research not involving greater than minimum risk.

Subjects:

Gender: Female

Age: Adult (18-64 years)

Ethnicity: All ethnicities

Primary Language: English, Spanish, Vietnamese

Groups to be Recruited: Asymptomatic patients with chronic conditions, healthy

Vulnerable populations: No

Pregnant woman/fetus: No

Neonates: No

Children: No

Design/Procedure

Research Category: Randomized effectiveness Study

Research Design:

Eligible women will be identified monthly through a query of the EMR database. Data will be extracted and transferred to OnCore (Forte Research Systems, Madison, WI) for randomization, storage, and data management. A computer-generated permutated block randomization scheme will be used to randomly assign individuals to the three trial arms with a 1:1:1 ratio using participants' medical record number. Randomized women will receive a study-specific identification number. Randomized women will be contacted by telephone. Those who are not reached will be contacted on 3 different days at 3 different times before being classified as unreachable. Unreachable participants will be replaced by randomly-selecting additional eligible women until the target sample size is met. Women who hang up the telephone before the scripted message is delivered will receive a second call within 10-30 minutes. Those who do not hear the full message on the second attempt will be categorized as refusers.

The 3 arms of the RCT are: Arm 1: Telephone recall (control). Participants will receive a scripted telephone recall from a trained patient navigator (PN) on behalf of Harris Health. Arm 2: Telephone recall with mailed HPV self-sampling kit (intervention). Participants in this arm will receive a scripted telephone recall from a PN on behalf of Harris Health and receive a mailed HPV self-sampling kit with a pre-paid return envelope.

Arm 3: Telephone recall with mailed HPV self-sampling kit and patient navigation (intervention plus). Participants in this arm will receive the same scripted telephone recall and mailed self-sampling kit as in Arm 2. Within 3-5 days of the kit's mail-out, participants will receive a telephone call from the PN.

Inclusion Criteria:

Randomized Controlled Trial. To be eligible for randomization, women must 1) be age 30-65 years; 2) have no history of hysterectomy or cervical cancer; 3) have at least 2 visits to ambulatory care within Harris Health System in the past 5 years; 4) be enrolled in a healthcare coverage or financial assistance plan accepted by Harris Health or have been enrolled in a Harris Health coverage plan in the past 12 months; 5) no have a history of cervical dysplasia in the past 3.5 years; and 6) have not had a Pap test in the past 3.5 years or Pap/HPV co-test in the past 5.5 years. A 6-month grace period is added to allow time for women to respond to opportunistic usual care strategies (i.e., in-clinic EMR-flagging and video-based patient education).

For analytical sub-aim 1 (Describe characteristics of women who under-attend for cervical cancer screening in a safety net health system), inclusion criteria are broadened to include all women who meet inclusion criteria 1-4, but not criteria 5 (i.e., women of all screening status will be included in the analysis).

Telephone Survey. To be eligible for the telephone survey, women must 1) have participated in RCT (and thus have met RCT inclusion criteria above) and 2) have been randomized to Arm 2 or 3.

Qualitative Interviews To be eligible for the qualitative interviews, women must 1) have participated in RCT (and thus have met RCT inclusion criteria above); 2) have been randomized to Arm 2 or 3; and 3) had an HR-HPV positive test result.

For COVID-19 era analytics sub-aims, inclusion criteria are broadened to include women ages 18-65 years who meet inclusion criteria 3 and 4 (i.e., we will include all women 18-65 years who have used

Harris Health ambulatory care services at least twice in the past 5 years and who have a healthcare coverage plan accepted by Harris Health.

Exclusion Criteria:

Randomized Controlled Trial Patients who self-pay will be excluded unless they were covered under a Harris Health healthcare coverage plan within the past 12 months. This is to ensure that all participants are able to receive follow-up clinical care from Harris Health System, as needed. For those with expired eligibility, expedited emergency coverage can be instituted as needed, per a process worked out with Harris Health System financial assistance leadership. Additional exclusion criteria will be assessed by chart review and at the time of attempted telephone contact. Specifically, we will exclude women who lack valid telephone contact information; are unable to communicate in English, Spanish, Vietnamese or other Asian language where translation services are available; or self-report being currently pregnant.

Telephone Survey: Women who test positive for HR-HPV and require clinical follow-up are not eligible for the survey.

Qualitative Interviews: Women who test negative for HR HPV are not eligible for the qualitative interviews.

Procedure:

Procedures for the three arms of the RCT are as follows: Arm 1: Telephone recall (control). Participants will receive a scripted telephone recall from a trained patient navigator (PN) on

behalf of Harris Health. PNs will let the patient know of the telehealth options available to Harris Health patients during the COVID-19 pandemic. Patients will be given the contact information for the call center that can walk them through the steps of accessing telehealth. PNs will also inform participants that their records indicate that they are overdue for a Pap test and that primary care, including well woman exams and Pap testing is still important during the pandemic. Arm 2: Telephone recall with mailed HPV self-sampling kit (intervention). Participants in this arm will receive a scripted telephone recall from a PN on behalf of Harris Health and receive a mailed HPV self-sampling kit with a pre-paid return envelope. PNs will state that they are calling on behalf of Harris Health, inform participants that their records indicate that they are overdue for a Pap test, instruct them to call the scheduling department to make an appointment, and provide the scheduling department's telephone number. PNs will also state that, "as an alternative to Pap test screening, you will receive a kit in the mail that allows you to collect your own sample for cervical cancer screening and send it back to the lab for testing." PNs will confirm the patient's address and within three business days, will mail participants the self-sampling kit via the US postal service. Completed kits will be returned to Harris Health lab for HR-HPV testing (described below). Arm 2 will also receive information on telehealth options available for Harris Health patients and will be given the phone number for help accessing telehealth. Arm 3: Telephone recall with mailed HPV self-sampling kit and patient navigation (intervention plus). Participants in this arm will receive the same scripted telephone recall and mailed self- sampling kit as in Arm 2. Within 3-5 days of the kits mail-out, participants will receive a telephone call from the PN. The PN will provide one-on-one education involving three overlapping domains: 1) information on the nature and purpose of cervical cancer screening and the causative role of HR-HPV; 2) the ability to complete screening through a clinic- based Pap test or through self-sample HPV testing using the kit; and 3) instruction on how to use and return the completed kit. Participants who indicate preference to undergo a Pap test will be assisted with scheduling an appointment with a Harris Health provider. Participants who indicated intent to self-sample but

whose kit is not received by the lab within 3 weeks of mail-out will receive up to 3 telephone reminders and a letter if unreachable by phone. Arm 3 will also receive information on telehealth options available for Harris Health patients and will be given the phone number for help accessing telehealth. Mailed self-sample HPV testing (Arms 2 and 3) HPV self-sampling kits will include an introductory letter, research information sheet, self-sampling kit, instructional brochure, and a labeled, pre-paid envelope addressed to the Harris Health central offices.

The introductory letter, on behalf of Harris Health, will invite women to use the self-sampling kit as part of a research study to evaluate new strategies for cervical cancer screening. The letter will ask women to complete the self-testing HPV kit as soon as they get the package and return their sample in the mail. The letter will refer patients to the research information sheet, which provides the telephone number for the Harris Health scheduling department, indicates that, as an alternative to a Pap test, participants can complete and return the enclosed self- sampling kit. The research information sheet will describe the study's purpose, procedures, voluntary participation, risks and benefits, and protection of privacy and confidentiality. It will provide a number to call to revoke authorization for review of medical record data and to report injury or other adverse events. The instructional brochure provides illustrated step-by-step instructions in English and Spanish and is written for comprehension at a fourth-grade reading level (see Section Z for attachments in English and Spanish). The self- sampling kit is the commercially-available Aptima Cervical Specimen Collection and Transport Kit (see Section Z for manufacturer's package insert). The kit consists of an individually-wrapped cervical swab and a vial of Aptima Specimen Transport Medium. Laboratory testing for HR-HPV using self-sampling kits: The vial of specimen transport medium will returned within a sealed biohazard bag (pre-labeled with the participant ID), which will be placed within a postage-paid padded return envelope, addressed to central Harris Health offices. The Aptima specimen transport vial is approved for shipping via standard mail. The vial

will then be routed by PNs to the central CLIA-certified laboratory at Harris Health. HPV testing on self-sampled kits will be conducted using the FDA-approved Aptima® HPV test (Hologic), which is the same assay used for standard of care clinical HPV testing. Aptima tests for 14 high-risk HPV types, including HPV 16 and 18. Per routine standard of care, HPV-positive samples will be reflex-genotyped for HPV 16 and 18/45 (the genotypes associated with 80% of invasive cervical cancers worldwide) to improve risk stratification and determine the appropriate algorithm for clinical follow-up. As is standard, test results will be interpreted as 1) HR-HPV negative, 2) HR-HPV positive and HPV 16/18/45 negative; 3) HR-HPV positive and HPV 16/18/45 positive; or 4) inadequate (due to unsatisfactory sample). Kits that are returned after >30 days will also be considered inadequate. Test results will be sent via Harris Health secure transmission to co-investigator Dr. Chiao, the study coordinator, and the PN team. Notification of test results and referral for clinical follow-up: HR-HPV positive test results will be communicated on a weekly basis to co-investigator Dr. Chiao. Dr. Chiao will be responsible for referring HR-HPV positive women to clinical follow-up. Notification to participants of both negative and positive results will be conducted by the PNs, who will contact participants by telephone within 10 days of the laboratory's receipt of the sample. Participants who are unreachable after 3 telephone attempts on different days/times will be mailed their results by certified mail.

Results will be reported as follows: HR-HPV negative women will be told that no high-risk HPV strains were found in their sample. However, since self-sampling is not currently approved for primary screening, women will be advised to attend for clinic-based screening within the next 12 months. Dr. Chiao is now affiliated with MD Anderson and will continue to provide clinical oversight for the trial and review lab results through the secure Harris Health EMR system. MD Anderson has been added as a site for this study with the BCM IRB. HR-HPV positive. Women

who test positive for HR-HPV but negative for 16/18/45 will be referred by Dr. Chiao to clinic-based screening (Pap/HR-HPV co-testing). They will be asked to make an appointment with their healthcare provider and given clinic contact information. Women who test positive for HR 16/18 will be referred to colposcopy. As is standard practice at Harris Health when screening abnormalities are encountered, women requiring follow-up Pap test screening or colposcopy who have not made an appointment within 30 days of test result notification will receive a telephone call from a PN who will assist them with scheduling an appointment. Inadequate: Women whose samples were inadequate (due to unsatisfactory sampling or kit returned after >30 days) will be advised to attend for clinic-based screening or mailed a new kit.

Ascertainment of Outcomes

Primary outcome. The primary outcome is primary screening participation, defined as completion and return of a mailed self-sample HPV testing kit that is tested in the lab or attendance for clinic-based screening within 6 months of randomization. Receipt of mailed self-sample HPV testing kits will be ascertained by reviewing lab records. Attendance for clinic-based screening will be ascertained by the study coordinator based on review of the Harris Health EMR. Primary screening participation will be dichotomously categorized as screened/unscreened. Secondary outcomes are screening tests results (positive, negative, or inadequate) and completion of clinical follow-up among women with an abnormal screening test result (attended, did not attend). Screening test results will be ascertained within 6 months of randomization. Pap/HR-HPV co-testing results will be ascertained by EMR review. Self-sample HR-HPV test results will be based on laboratory reports.

Completion of clinical follow- up will be ascertained by EMR review within 6 months of the date of the screening test result. Completion of clinical follow-up is defined as attendance for colposcopy among participants who had a positive test by clinic-based screening and attendance for colposcopy or subsequent clinic-based screening among those who had a positive test by self-sampling. Additional exploratory outcomes (sub-aim 2) are detection and treatment of cervical precancers (i.e., histologically-confirmed cervical intraepithelial neoplasia grade II or greater [CIN2+]). Precancers will be ascertained by EMR review within 6 months of abnormal screening results and treatment as per ASCCP guidelines will be ascertained within 6 months of the date of diagnosis. Completion of primary care appointments will be ascertained at 6 months, including telehealth appointments.

Additional variables will be collected to meet the aims of the NIH-funded supplement (3R01MD013715-04S1) to the parent trial: HIV status, Preventive Care Gap Score, Enrollment in MyHealth portal, History of COVID-19 diagnosis, HPV vaccination status and Risk of Admission or ED visit. We will also collect Mental and Behavioral Disorders due to use of the following: alcohol (Code F10), opioids (Code F11), cannabis (Code F12), sedatives, hypnotics, anxiolytics (Code F13), cocaine (CodeF14), other stimulants, including caffeine (Code F15), hallucinogens (Code F16) nicotine (Code F17), inhalants (Code F18) and other psychoactive substances and multiple drug use (Code F19). Data collection instruments are attached in Section S: Attachments, file name: PRESTIS Forms." Instances of Injury and Adverse Events (AE) will be handled as follows. In the Research Information Sheet, participants will be informed of the risks of using the self-collection device and swab. The sheet will include a definition and examples of adverse events, as well as phone numbers to report an AE to the study team and to make an appointment with a Baylor OB/GYN (see RIS in English/Spanish in Section Z) Participants are considered "on-study" through ascertainment of primary and secondary outcomes

described above. While we do not anticipate AEs after the "on-study" period, AEs will be monitored for 90 days after participants go "off-study. Community Advisory Board: We will work with various community partners to serve on the project's community advisory board (CAB). Materials and patient navigator scripts are still being translated from English to Vietnamese by a professional translator and will be submitted via an amendment in BRAIN for IRB review and approval prior to implementation. Facilitated by Ms. Nguyen, CAB members will review the documents for linguistic and cultural appropriateness and plain language.

Sample Size/Data Analysis

Sample Size: Local: 2,508; Worldwide: 2,508

Sample Size Justification:

Randomized Controlled Trial: Calculations were based on a conservative estimate that mailed self-collection would yield a 6% increase in cervical cancer screening participation compared to usual care (estimated at 18%). In a systematic review, patient navigation was associated with a 15% improvement over standard of care for completion of primary colorectal cancer screening, with the proportion of participants who complete screening after navigation ranging from 27-67%.. As the degree to which HPV and colorectal screening are analogous is unknown, we chose to err on the conservative side and, in order to ensure adequate power, based our calculations on the assumption that at least a 6% difference exists between screening non-attendees who complete mailed self-sample HPV testing with patient navigation compared to those who complete the self-sample testing without patient navigation. With 756 participants in each study arm and a total of 2,268 (nQueryAdvisor version 7.0), we can expect to detect the indicated differences in proportions between Arms 2 and 3. Given the large numbers of screening non-attendees enrolled in Harris

Health (>15,000), we anticipate that we will be able to accrue this number of participants over a period of 30 months, with completion of follow-up in 36 months.

The sample size has been revised to account for an additional 240 Asian/Asian American women who will be enrolled as part of an administrative supplement. For the Asian/Asian American targeted enrollment study, the estimated number of participants who can be enrolled in a six month period with the two patient navigators who will remain on the team is 240. A recent meta-analysis of trials showed a pooled 12.8% improvement in intent-to-treat analyses measuring screening uptake in mail-to-all self-sample HPV testing compared to usual care. Using this data, our sample size of 240 (120 in Arm 1, 60 each in Arms 2 and 3) provides 85.2% power to detect an improvement of 12.8% between usual care (Arm 1) and mailed self-sampling (Arms 2 and 3 combined) with alpha=0.05 (30).

Telephone Survey: The sample size is designed to assess the main effect of patient navigation on reported barriers to self-sampling. Specifically, it is designed to detect a 25% difference in the proportion of participants who report one of the top three barriers reported in our previous research (concerns of injury, safety, and cleanliness), comparing women who did and did not receive patient navigation. Specifying an expected proportion of 0.30, 80% power, and a=0.05, the target sample size is 272 (n=68 in each group of the factorial design).

For the Asian/Asian American targeted enrollment study, we will conduct an additional 40 telephone surveys among a random sample of Asian/Asian American participants in the self-sampling arms (Arms 2 and 3) who do (n=20) and do not (n=20) return their self-sampling kit.

Qualitative Interviews: We will invite 15 HR-HPV positive women who attend and 15 who do not attend for clinical follow-up within 6 months of their test results. Based on our pilot data, we estimate that this number of participants will be sufficient to reach data saturation (i.e., redundancy in results to the point where additional interviews do not provide new information) and allow us to identify themes and trends in the data. Participants will be purposively selected to attain a demographically diverse sample.

We will conduct 10 semi-structured telephone interviews with 10 Asian/American participants who complete self-sample HPV testing and who test positive for HR-HPV to identify reactions to a positive test and attitudes and barriers to clinical follow-up.

Data Analysis:

Randomized Controlled Trial: Primary outcome. Primary screening participation will be examined dichotomously (screened/not screened) using an "intent-to-screen" analytic approach. Bivariable tables and Pearson's x2 tests will be used to compare the proportion of primary screening participation across Arms 1, 2, and 3, as well as the absolute difference in participation across Arms 1, 2 and 3. Bivariable tables and Pearson's x2 tests will be used to compare the proportion of primary screening participation by study arm, as well as the absolute difference in participation across arms. Log binomial regression will be used to calculate the relative risks of primary screening participation and corresponding 95% confidence intervals (Cls). Secondary outcomes. Descriptive analyses of secondary outcomes will be conducted using bivariable tables and Fishers exact tests to describe and compare test results and attendance for clinical follow-up across study arms 1, 2 and 3. Log binomial regression will be used to calculate relative risks and 95% Cls of having a positive screening test and of attending for clinical follow-up.

Analytic sub-aim: 1) We will use descriptive statistics (bivariable tables and chi-square/Fisher's exact tests) to compare characteristics of un/under-screened women and those who are up-to-date on screening across Arms 1, 2, and 3. Multivariable logistic regression will be used to assess the independent association between screening status and participant characteristics. 2) Detection and treatment of precancers will be explored through descriptive analyses using bivariable tables and Fisher's exact tests. 3) Among participants in Arms 1, 2 and 3, we will conduct separate analyses to assess characteristics associated with primary screening participation. For Arms 1, 2 and 3, we will use bivariable tables with Pearson's x2 tests to describe characteristics of screened and unscreened participants. Characteristics of interest include age, race/ethnicity, months since last Pap test, health plan type, number of healthcare encounters in the past 12 months, and time enrolled as a Harris Health patient. Associations with $p<0.10$ will be used to determine candidate variables for subsequent multivariable models. Multivariable log binomial regression models will be built to assess the independent association between screening participation and participant characteristics after adjusting for relevant covariates. The likelihood ratio test will be used to examine the relative contribution of specific variables to the fit of the model. Adjusted risk ratios will be reported and will be considered statistically significant at $p<0.05$.

Telephone Survey: Descriptive statistics will be used to identify experiences of self-sampling among survey participants who completed and returned a kit. The main effects of patient navigation on overall experience will be assessed using bivariable tables and Pearson's x2 or Fisher's exact tests. Associations with $p<0.05$ will be considered statistically significant. Additionally, we will explore patterns related to specific barriers in the scale using exploratory bivariable analyses. For those participants who did not return a kit, descriptive statistics will be used to describe barriers to clinic-based screening and reasons for using the self-sampling kit.

Bivariable tables and Pearson's x2 or Fisher's exact tests will be used to explore differences in barriers to clinic-based screening between kit returners and non-returners.

Qualitative Interviews: The semi-structured interviews will be transcribed by a professional transcription service and kept in the language in which they were conducted (English or Spanish). The Pl, who is bilingual in English and Spanish and experienced in qualitative data analysis, will conduct a close reading of the transcripts to develop a coding scheme. Once the coding scheme is developed, coding rules and procedures will be detailed in a codebook and described to two independent coders (the research coordinator and research assistant) who will classify the dataset. Cohen's Kappa coefficient [86] will be used to evaluate inter-coder reliability. Iterative content analysis will be used to collapse codes into emergent themes. A summative approach will be used to describe and compare codes and themes across women who do and do not attend for clinical follow-up.

COVID-19 era analytic sub-aim: 1) We will use descriptive statistics (bivariable tables and chi-square/Fisher's exact tests) to compare characteristics of women who do/do not attend for cervical cancer screening, primary care (all types), and telehealth-specific primary care. We will compare proportions at baseline (February 2020, pre-pandemic), and at 6- and 12-months post COVID-19 emergency declaration (July 2020 and February 2021). Independent multivariable Poisson regression models will be used to assess the independent association between cervical cancer screening and primary care utilization and participant characteristics.

Additional variables will be collected to meet the aims of the NIH-funded supplement (3R01MD013715-04S1) to the parent self-sampling trial: HIV status, Preventive Care Gap

Score, Enrollment in MyHealth portal, History of COVID-19 diagnosis, HPV vaccination status and Risk of Admission or ED visit.

Potential Risks/Discomforts

Randomized Controlled Trial: For participants in the control arm (Arm 1) there are mild emotional risks from learning or being reminded of being past-due for recommended cervical cancer screening. Additionally, there is risk of potential loss of confidentiality.

For participants in the intervention arms (Arms 2 and 3), there are mild emotional risks from learning or being reminded of being past-due for recommended cervical cancer screening. There are minor physical risks of using the self-sampling device. Specifically, the self-sampling device may be a somewhat uncomfortable to use and may cause a very low level of pain and minor bleeding (spotting) during and/or after use that is caused by a small scratch to the vagina or cervix. These risks are minimal with no such events being reported in previous studies. In the Research Information Sheet, we provide subjects with clear instructions about how to manage these potential but rare adverse events. In the case of pain and bleeding, the symptoms should resolve on their own within a few days.

In the Research Information Sheet, participants are told that pregnant women should not use the collection device. In the event that they find out later that they were pregnant when they used the device, they are instructed to inform their OB/GYN and provided the number for BCM OB/GYN if they do not have an OB/GYN.

The Aptima transport medium is non-hazardous and non-flammable. Detailed instructions for safely returning the vial to the laboratory are described in the instructional brochure. After the vial has been tightly capped, participants are instructed to place the capped vial into the provided biohazard bag and seal the bag using the Ziploc mechanism. In accordance with shipping rules, this inner receptacle (biohazard bag) must have an inner or outer absorbent material capable of absorbing the volume of the liquid. Thus a Green Z Drop-in Pac will be included within each biohazard bag. This will ensure that any inadvertent spillage during shipping will be absorbed.

There are potentially emotional risks of testing for HPV, especially among women who learn they are positive for high-risk HPV. There is also a small chance that individuals who test negative for high-risk HPV are in fact infected. Finally, there is a risk of potential loss of confidentiality.

*While the study involves only minimal risk, the funding agency requested a Data Safety Monitoring plan (attached in Section S).

Telephone Survey There are mild emotional risks from participating in the survey, as some questions inquire about barriers to access to care and cervical cancer screening. There is risk of potential loss of confidentiality.

Qualitative Interviews There are mild emotional risks from participating in the semi-structured interviews, as some questions inquire about barriers to access to care and diagnostic follow-up, as well as emotional distress caused by an HR-HPV positive test result. However, in common practice, women usually benefit from talking about the emotional distress caused by a positive cancer screening test. There is risk of potential loss of confidentiality.

Data and Safety Monitoring Plan

Do the study activities impart greater than minimal risk to subjects? No

Coordination of information among sites for multi-site research: BCM is the Coordinating Center for this multi-site study. The partner sites listed below will rely on BCM IRB (reliance has been reached through SMART IRB). All administrative, research and other trial-related activities, including reporting of unanticipated problems, protocol modifications, IRB and/or institutional approvals and interim results, will be directed by and based at BCM. All of the research records and research related information will be retained at BCM. All data is stored in a password-protected BCM Box account, which is within Baylor's firewall-protected server.

MD Anderson Cancer Center- Dr. Chiao will provide clinical oversight for the trial, and will review trial participants' lab results. Additionally, the Pl of the grant, Dr. Montealegre will be provided access to the data through a Data Transfer Agreement between Baylor College of Medicine and MD Anderson. University of South Florida- Dr. Matt Anderson will be involved in the interpretation of study data.

All administrative, research and other trial-related activities will be directed by and based at BCM. All of the research records and research related information will be retained at BCM. After the execution of a Data Transfer Agreement, trial data will be shared with the Pl of the grant and co-investigators at MD Anderson Cancer Center who are involved in data analysis and interpretation of study results.

Potential Benefits

Describe potential benefit(s) to be gained by the individual subject as a result of participating in the planned work.

Randomized Controlled Trial All participants will directly benefit from the study by receiving an intervention to facilitate their completion of primary cervical cancer screening. Specifically, participants in Arms 1 will receive an evidence-based telephone recall intervention known to improve participation in clinic-based screening. Participants in Arms 2 and 3 will also receive a telephone recall intervention and may benefit from completing a self-sample HPV test kit and receiving their results. If found positive for high risk-HPV, participants in Arms 2 and 3 will be navigated to a Harris Health community health center for clinical follow-up, as is standard practice at Harris Health System when screening abnormalities are detected.

Telephone Survey Some individuals may perceive a personal benefit of "debriefing" after using the self-sampling kit and from having the opportunity to express potential concerns. There are no other direct benefits to participating in the survey.

Qualitative Interviews Some individuals may perceive that they benefit from discussing the emotional impact of a positive cancer screening test. There are no other direct benefits to participating in the interview.

Describe potential benefit(s) to society of the planned work.

Long-term, society will benefit from the insights generated by the proposed study regarding screening strategies that can be used to reach women who are otherwise unable or unwilling to

participate in standard of care cervical cancer screening. The results of this study are expected to lay the groundwork for creating models of care to reach women who otherwise do not participate in standard of care screening. Long-term, this model can be adopted and scaled to eliminate cervical cancer screening disparities and reduce the burden of cervical cancer-related disease.

The telephone survey and qualitative interviews will provide crucial data regarding acceptability and experiences of self-sample HPV testing which can be used to improve the intervention in the future. The qualitative interviews will additionally inform emotional and information needs of women who have a positive HR-HPV test.

The COVID-19 era analytic sub-aims will elucidate how the pandemic affected use of cervical cancer screening and primary care services in a safety net population.

Do anticipated benefits outweigh potential risks? Discuss the risk-to-benefit ratio.

We believe these potential benefits measurably outweigh any potential emotional distress, minor physical harm and small risk of loss of confidentiality posed by the trial and participation in the telephone survey and qualitative interviews.

Consent Procedures

Will any portion of this research require a waiver of consent and authorization? Yes

Please describe the portion of the research for which a waiver is required.

We are requesting a waiver of consent to conduct the chart review to determine subject eligibility. We are also requesting a waiver of consent for subjects in Arm 1 (control arm). Telephone recalls are standard care in many health systems; additionally: 1) participation involves no more than minimal risk; 2) the waiver will not affect the rights and welfare of participants; and 3) the research could not practically be done without the waiver. Finally, we are requesting a waiver of consent from subjects in Arms 2 and 3 (intervention arms) who do not complete and return the self-sampling kit. Even though they do not participate in the intervention, we would like to review their medical record to determine if they subsequently attended for clinic-based screening. The following criteria are met: 1) participation involves no more than minimal risk; 2) the waiver will not affect the rights and welfare of participants; and 3) the research could not practically be done without the waiver.

Explain why the research and the use or disclosure of protected health information involves no more than minimal risk (including privacy risks) to the individuals.

Chart review to determine eligibility: The proposed chart to identify patients who are eligible for participation in the RCT involves only minimal risks to individuals. The risks to privacy are small and the waiver will not adversely affect the patients' privacy rights or welfare. While PHI will be recorded during the chart review, the the database will be stripped of patient identifiers for data analysis.

Subjects enrolled in Arm 1 (control group): Use of PHI for subjects in this groups involves minimal risk. Participants in the control arm receive standard-of-care services (patient recalls) that are already used at Harris Health (based on availability of grant funding) to encourage screening participation among un-/underscreened women. Screening outcomes will be assessed by chart

review for participants in this group, but this is not different to what would be done for routine program evaluation purposes.

Subjects enrolled in Arms 2 and 3 (intervention groups): Use of PHI for subjects in this group involves minimal risk. These are participants who declined the self-sampling intervention but may still feel encouraged to participate in standard-of-care clinic-based screening. Screening outcomes will be assessed by chart review.

Explain why the waiver will not adversely affect the privacy rights and the welfare of the research subjects.

The risks to privacy are small and this waiver will not adversely affect the patients' privacy rights or welfare. PHI will be collected to assess eligibility for participation and to determine screening outcomes among control group participants and intervention group participants who decline to use the self-sampling kit. Once these chart reviews are complete, the dataset will be stripped of PHI. Data will be securely stored on password-protected database servers,

Explain why the research could not practicably be conducted without the waiver and could not practicably be conducted without access to and use of the protected health information.

Waiver to determine subject eligibility: There are over 100,000 women who are age-eligible for cervical cancer screening within Harris Health System. Individually contacted them and seeking consent to determine their eligibility for the study (based on their cervical cancer screening status) would be practically impossible.

Waiver for subjects enrolled in Arm 1: Requiring informed consent for a standard-of-care intervention, such as patient recall, would introduce substantial bias in terms of participation in the intervention. Ascertaining screening outcomes related to this standard-of-care intervention cannot be done without accessing and using PHI.

Waiver for subjects enrolled in Arms 2 and 3: Ascertaining screening outcomes cannot be done with accessing and using PHI. Individually contacting each participant to obtain consent for this review is not feasible.

Describe how the research could not practicably be carried out without using the collected identifiable biospecimens in an identifiable format.

The samples collected will need to have identifiable information in order to link the specimen to patient's data in the electronic medical record.

Describe how an adequate plan exists in order to protect identifiers from improper use and disclosure.

Only study staff specifically involved in this study will have access to the database. Data will be stored on a password-protected database on the secure BCM server. Once the chart review is complete, the dataset will be stripped of PHI. Only aggregate data will be reported.

Describe how an adequate plan exists in order to destroy identifiers at the earliest opportunity consistent with conduct of the research, unless there is a health or research justification for retaining the identifiers or such retention is otherwise required by law.

Access to data will be limited and based on the role of personnel. Study personnel will only use necessary data and will make every effort to minimize risk and protect confidentiality.

Describe how adequate written assurances exist in order to ensure that the PHI will not be reused or disclosed to (shared with) any other person or entity, except as required by law, for authorized oversight of the research study, or for other research for which the use or disclosure of the PHI would be permitted under the Privacy Rule.

Sample receipt and subsequent test results will be recorded per standard of care in the patient's protected Harris Health electronic medical record. The study coordinator will securely access the Electronic Medical Record to extract relevant research data (sample receipt and outcome). The extracted research information will be stored on secure, password- protected database on BCM servers. These servers are physically located in the BCM data center and inside the BCM firewall. Servers are managed by BCM DLDCCC informatics professionals in accordance with BCM guidelines.

Will additional pertinent information be provided to subjects after participation? If No, explain why providing subjects additional pertinent information after participation is not appropriate: No

Given the parallel accrual of participants to each arm of the study and the long follow-up time needed to ascertain screening outcomes among participants randomized to each arm, we do not have sufficient time in the project period to cross-over control group participants to either of the intervention arms. We thus do not think it is appropriate to give control group participants information of self-sample HPV testing, which is not regularly available at Harris Health. However, all participants, including those in the control arm, will receive some type of intervention

(specifically patient recall in the control arm), which is expected to increase their participation in cervical cancer screening.

Waiver of requirement for written documentation of Consent

Will this research require a waiver of the requirement for written documentation of informed consent? Yes

Explain how the research involves no more than minimal risk to the participants, and the specifics demonstrating that the research does not involve procedures for which written consent is normally required outside of the research context.

Randomized Controlled Trial Participation in the trial involves only minimal risk to participants. Testing for HR-HPV has been shown to be equally or more sensitive than liquid-based cytology (i.e., Pap testing) for detecting invasive cervical cancer and high-grade lesions. Reflecting its sensitivity, HPV testing using provider-collected samples is now recommended by the US Food and Drug Administration (FDA) as an alternative to Pap test screening. Recently, it has become the primary screening tool in The Netherlands and Australia and is being considered as such in several other countries. Substantial evidence indicates that samples can be collected by providers or by women themselves, with similar sensitivity for detecting HPV nucleic acid. Potential adverse events (AEs) associated with the self-sampling intervention are comparable to those encountered by women undergoing clinic-based screening. Expected AEs are discomfort, minor pain, and (rarely) light bleeding. These will be captured through self-report to the study telephone number, which will be regularly monitored by study staff and reported to the IRB. AEs will be monitored

while participants are "on-study" (through ascertainment of primary and secondary outcomes) and for 90 days after participants go "off-study."

Other minimal risks include risks regarding loss of confidentiality. However, we will take every precaution to ensure that confidentiality is maintained at all times.

There is precedent for seeking a waiver of documented consent for this type of research. As described in Section C, there have been multiple large-scale pragmatic trials evaluating how self-sample HPV testing can be integrated within cervical cancer screening programs to increase screening rates among women who otherwise do not or under-attend for clinic- based screening. These trials have been conducted as part of national screening programs in Europe, Australia, and Canada. In the U.S., mailed self-sample HPV testing has been evaluated in the HOME (Home-based Options to Make Cervical Cancer Screening Easy) trial, a pragmatic randomized controlled trial within the Kaiser Permanente system. In all of these trials, the research has relied on a waiver of consent or waiver of documented consent in order to evaluate the real-world implementation of this intervention within an organized screening program.

Rather than a formal informed consent form, we will use a Research Introduction Letter and Research Information Sheet which contains all the information usually contained in a consent form. We request not to require signing the form, however, because we fear that it will introduce significant bias into our research. Instead we have included language in both the letter and the information sheet that makes it clear to participants that their completion and return of the self-sampling kit indicates their consent to participate.

Telephone Survey and Qualitative Interviews We are seeking a waiver of written consent for the Telephone Survey and Qualitative interviews. We will use an informed consent script that will be read to participants over the telephone. Once they have been read the script and given the opportunity to ask questions, participants will be asked to verbally indicate whether the consent to participate (see Section S: Consent Script for Telephone Survey and Consent Script for Qualitative Interviews). Because the surveys and interviews are conducted over the telephone, rather than in person, it is impractical to have a physical informed consent form that needs to be signed. Mailing the informed consent form to participants home would severely decrease participation and likely lead to a biased survey.

Consent Procedures

Describe how research population will be identified, recruitment procedures, any waiting period between informing the prospective participant and obtaining consent, steps taken to minimize the possibility of coercion or undue influence and consent procedures in detail.

Randomized Controlled Trial Eligible women will be identified monthly through a query of the EMR database. Data will be extracted and transferred to OnCore (Forte Research Systems, Madison, WI) for randomization, storage, and data management. A computer-generated permutated block randomization scheme will be used to randomly assign individuals to the three trial arms with a 1:1:1 ratio using participants' medical record number. Randomized women will be contacted by telephone. We have requested a waiver of consent the data extraction and phone calls. Patients who are not reached will be contacted on 3 different days at 3 different times before being classified as unreachable.

The patient navigators performing the calls will undergo training in Human Subjects research and arm-specific procedures. The language used by the patient navigators is standard to non-research patient navigation, and is designed in a way that makes it clear to patients that they are free to make their decisions of whether or not they would like to participate in screening.

For patients in Arm 2 and 3, we will use an Introduction Letter and Research Information Sheet which contains all the information usually contained in a consent form rather than a formal informed consent form. The Introduction Letter indicates that, as an alternative to a Pap test, participants can complete and return the enclosed self-sampling kit. It clearly states that the self-sampling kit is part of a research study conducted by Baylor College of Medicine. It also asks participants to closely read the attached Research Information Sheet. The Research Information Sheet describes the study's purpose, procedures, voluntary participation, risks and benefits, and protection of privacy and confidentiality. Both documents The Research Information Sheet states that participation is voluntary and that choosing not to participate does not result in any lost rights or benefits as a Harris Health patient. Potential participants are also informed that they may decide to stop taking part at any time and that doing so will not change the health care that they receive now or in the future. The letter provides a number to call to revoke authorization for review of medical record data. It also includes a number to call with questions and concerns and to report an injury or other adverse event

We request not to require signing the form, because we fear that it will introduce significant bias into our research. Instead we have included language in both the letter and the information sheet that

makes it clear to participants that their completion and return of the self-sampling kit indicates their consent to participate.

Finally, none of the subjects are being compensated for their participation in this study, so there is no financial influence in participant's decision to take part in the study. The introductory letter and research information sheet are available in English and Spanish. Both versions are attached in Section S.

Telephone Survey: Women who are randomly selected to participate in the telephone survey and women who are selected to participate in the qualitative interviews will be contacted by telephone by a trained, bilingual research assistant and invited to participate in the survey. The informed consent will be read to participants, emphasizing voluntary nature of the study and that participation will not affect their participation in the larger RCT or their ability to obtain healthcare services at Harris Health. Women will be given the opportunity to ask questions. After doing so, they will be asked to verbally indicated whether they agree to participate in the survey/interview.

Consent and Enrollment of Vietnamese-speaking Subjects See attachment, file name: "209230 - Baylor College of Medicine_COA" for certificate of translation of Vietnamese- language materials. See attachment, file name: "Consent and Enrollment of Vietnamese-speaking Subjects".

Who will recruit subjects for this study? Pl's staff

Are foreign language consent forms required for this protocol? Yes

Which of the following ways will you document informed consent in languages other than English?

A full-length informed consent document

Research Related Health Information and Confidentiality

Will research data include identifiable subject information? Yes

At what institution will the physical research data be kept? Physical research data will be kept in a locked cabinet in the Pl's office at BCM. Before mailing them out, labeled self-collection kits will be stored in the study office at BCM.

How will such physical research data be secured? Physical research data will be kept in a file cabinet that will be locked at all times. Only authorized study personnel and the Pl will have access to the data.

At what institution will the electronic research data be kept?

Electronic research data will be kept in a secure, password-protected BCM server. Only study personnel and investigators will have access to the data. PHI data, such as the test results of the testing kits will be stored in the patientls Electronic Medical Record at Harris Health per standard of care. Study coordinators will access the EMR to extract this data, which will then be stored on the password-protected BCM servers.

Will there be anyone besides the Pl, the study staff, the IRB and the sponsor, who will have access to identifiable research data?

Like other screening test results, non-study healthcare providers who have access to the electronic medical record will be able to access self-sample HPV testing results.

Through a Data Transfer Agreement, datasets will be shared with investigators at The University of Texas MD Anderson Cancer Center who will be involved with data analysis and interpretation. Specifically, the Pl of the NIH award and her team have moved from BCM to MD Anderson.

Please describe the methods of transmission of any research data (including PHI, sensitive, and non-sensitive data) to sponsors and/or collaborators.

Communication between study staff, patient navigators, and clinical co-investigators (Dr. Chiao and Dr., Zare, Medical Chief of Staff) that involves PHI will be conducted through OnCore, secure email, and secure BCM One Drive.

Through a Data Transfer Agreement (DTA), study datasets will be shared with co-investigators at The University of Texas MD Anderson. Transmission of data will be done securely in accordance with the rules and regulations of the Data Transfer Agreement. Secure BCM One Drive is currently used for transmission of PHI as stated above and can also be used for transmission of study datasets, if stipulated in the DTA.

De-identified patient trial data will be sent to investigators at MUSC. MUSC will not be sending data/specimens to BCM. Patient trial data including PHI will be sent to investigators at MDACC.

MDACC will not be sending data/specimens to BCM. De-identified patient trial data and de-identified specimens will be sent to Rice University. Rice U will not be sending data/specimens to BCM. Data will be shared via secure file sharing using password-protected BCM Box account, which is within Baylor's firewall protected server. De-identified data will be shared with MUSC investigators. Raw data will be shared with MDACC investigators.

A DTA and MTA will be obtained for data transfer between BCM and MDACC. The BCM Pl will contact BCM SPO to determine whether a DTA/DUA/MTA is required for the proposed data transfers with Rice University. These agreements will be established if determined necessary by SPO.

Cost/Payment

Delineate clinical procedures from research procedures. Will subject's insurance (or subject) be responsible for research related costs? If so state for which items subject's insurance (or subject) will be responsible (surgery, device, drugs, etc). If appropriate, discuss the availability of financial counseling.

Research procedures are those related to self-sample HPV testing (for participants in Arms 2 and 3 of the trial). Clinic-based screening and procedures used for clinical follow-up following an abnormal screening test (e.g., Pap test or colposcopy) are standard of care. As participants are by definition Harris Health System patients, these procedures will be covered according to the patient's health insurance/health coverage plan.

Sample Collection

SAMPLE: Other: cervical swabs

What is the purpose of the sample collection? Cervical swabs will be self-collected by participants and tested for high-risk HPV

Will the sample be stripped of identifiers? No

Will sample be released to anyone not listed as an investigator on the protocol? Will the information be identifiable, coded or de-identified?

The sample will not be released to other investigators who are not on the protocol.

Will sample material be sold or transferred to any third parties? Will the information be de-identified?

The sample material will not be sold or transferred to third parties.

Where will the sample be banked and for how long?

After laboratory testing at Harris Health, the PreservCyt vials will be transferred for storage at BCM, specifically in the DLDCCC Population Science Biorepository (directed by co- investigator Dr. Michael Scheurer). The samples will be banked indefinitely for future IRB-approved research (specifically for HPV testing and validation of HPV tests currently under development.

Does the banking institution have an approved policy for the distribution of samples? BCM is the banking institution and has an approved policy for storing specimens. Banked specimens will only be used by investigators listed on this protocol.

Will the remaining tissue be discarded? The remaining sample will be stored in the DLDCCC Population Sciences Biorepository. Samples will be kept indefinitely.

Will samples be made available to the research subject (or his/her medical doctor) for other testing? No

Will subject have the option to get the remaining portion of their sample back? No

Will samples be destroyed? If not, will they be kept anonymously? What will happen to the sample if the subject revokes authorization? The research information sheet informs subjects that they can revoke authorization for banking of their remaining specimen by calling the Pl. If authorization is revoked, the sample will be discarded.

Will data obtained from their sample be deleted? What will happen to the sample if the subject revokes authorization?

If subjects revoke authorization, their HPV test results will be deleted.

Will study data or test results be recorded in the subject's medical records? Yes

Will results of specific tests and/or results of the overall study be revealed to the research subject and or his/her doctor?

HPV test results will be recorded in subject's Harris Health medical records. These can be accessed by both the subject and her doctor.

Please identify all third parties, including the subject's physician, to receive the test results.

HPV test results will be recorded in subject's Harris Health medical records. These can be accessed by both the subject and her doctor. Providers involved in clinical follow-up (i.e., colposcopy or follow-up Pap) will also receive the results. Other providers with access to the electronic medical record can access test results, but will not be notified of them.
