# Supplementary material for: Self-Collection for Cervical Cancer Screening in a Safety-Net Setting: The PRESTIS Randomized Clinical Trial
Source: JAMA Intern Med. 2025 Jun 6;185(9):1119–27. doi: 10.1001/jamainternmed.2025.2971 (PMC12144659; doi:10.1001/jamainternmed.2025.2971)
Supplement: Supplement 2. — eMethods. Statistical Analysis Plan eFigure. Study outcomes: primary (screening participation), secondary (screening test results and attendance for clinical follow-up), and exploratory (diagnosis and treatment) [file jamainternmed-e252971-s002.pdf]

Supplemental Online Content

Montealegre JR, Hilsenbeck SG, Bulsara S, et al. Self-collection for cervical cancer screening in a safety-net setting: the PRESTIS randomized clinical trial. *JAMA Intern Med*. Published online June 6, 2025. doi:10.1001/jamainternmed.2025.2971

eMethods. Statistical Analysis Plan
eFigure. Study outcomes: primary (screening participation), secondary (screening test results and attendance for clinical follow-up), and exploratory (diagnosis and treatment)

This supplemental material has been provided by the authors to give readers additional information about their work.

© 2025 Montealegre JR et al. *JAMA Intern Med*.

eMethods. Statistical Analysis Plan

No separate Statistical Analysis Plan was used. The text below is from the Protocol.

Primary outcome. Primary screening participation will be examined dichotomously (screened/not screened) using an "intent-to-screen" analytic approach. Bivariable tables and Pearson's x2 tests will be used to compare the proportion of primary screening participation across Arms 1, 2, and 3, as well as the absolute difference in participation across Arms 1, 2 and 3. Bivariable tables and Pearson's x2 tests will be used to compare the proportion of primary screening participation by study arm, as well as the absolute difference in participation across arms. Log binomial regression will be used to calculate the relative risks of primary screening participation and corresponding 95% confidence intervals (CIs). Secondary outcomes. Descriptive analyses of secondary outcomes will be conducted using bivariable tables and Fishers exact tests to describe and compare test results and attendance for clinical follow-up across study arms 1, 2 and 3. Log binomial regression will be used to calculate relative risks and 95% CIs of having a positive screening test and of attending for clinical follow-up.

© 2025 Montealegre JR et al. *JAMA Intern Med*.

eFigure 1. Study outcomes: primary (screening participation), secondary (screening test results and attendance for clinical follow-up), and exploratory (diagnosis and treatment)

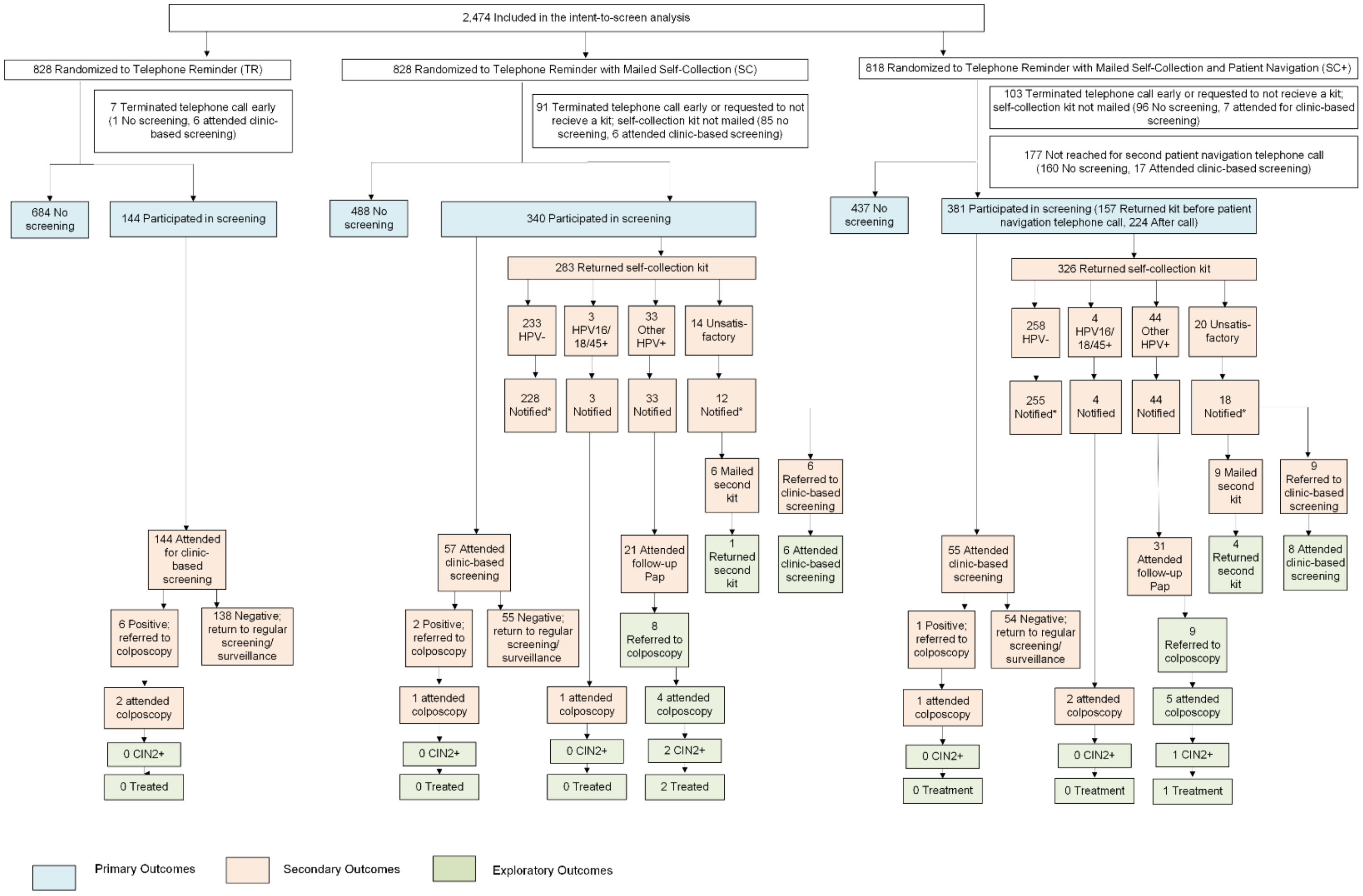

*Participants who were not notified could not be reached or declined the notification.

Abbreviations HPV: Human Papillomavirus; CIN 2+: Cervical Intraepithelial Neoplasia grade 2 or worse

© 2025 Montealegre JR et al. *JAMA Intern Med*.
