# Supplementary material for: Self-Collection for Cervical Cancer Screening in a Safety-Net Setting: The PRESTIS Randomized Clinical Trial
Source: JAMA Intern Med. 2025 Jun 6;185(9):1119–27. doi: 10.1001/jamainternmed.2025.2971 (PMC12144659; doi:10.1001/jamainternmed.2025.2971)
Supplement: Supplement 3. — Data Sharing Statement [file jamainternmed-e252971-s003.pdf]

Data Sharing Statement

Montealegre. Self-Collection for Cervical Cancer Screening in a Safety-Net Setting. *JAMA Intern Med*. Published June 06, 2025. doi:10.1001/jamainternmed.2025.2971

Data
Additional Information: ClinicalTrials.gov NCT03898167
Data available: Yes
Data types: Deidentified participant data
How to access data: Please send data requests to jrmontealegre@mdanderson.org
When available: With publication

Supporting Documents
Document types: None

Additional Information
Who can access the data: Researchers who provide a methodologically sound proposal
Types of analyses: To achieve the aims of the approved proposal,
Mechanisms of data availability: After approval of a proposal.
Any additional restrictions: None
